# Mitotic Phosphorylation of Swi6/HP1 Regulates Its Chromatin Binding and Chromosome Segregation

**DOI:** 10.1096/fj.202500384R

**Published:** 2025-11-02

**Authors:** Yuriko Yoshimura, Aki Hayashi, Mayo Tanaka, Mieko Suzuki‐Matsubara, Reiko Nakagawa, Gohei Nishibuchi, Hideaki Tagami, Masaya Oki, Jun‐ichi Nakayama

**Affiliations:** ^1^ Division of Chromatin Regulation National Institute for Basic Biology Okazaki Japan; ^2^ Department of Applied Chemistry and Biotechnology, Graduate School of Engineering University of Fukui Fukui Japan; ^3^ Graduate School of Science Nagoya City University Nagoya Japan; ^4^ Laboratory for Cell‐Free Protein Synthesis RIKEN Center for Biosystems Dynamics Research Kobe Japan; ^5^ Laboratory of Stem Cell Genetics, Institute for Life and Medical Sciences Kyoto University Kyoto Japan; ^6^ Basic Biology Program Graduate Institute for Advanced Studies, SOKENDAI Okazaki Japan

**Keywords:** fission yeast, heterochromatin, heterochromatin protein 1, mitosis, phosphorylation

## Abstract

In eukaryotic cells, heterochromatin assembly is critical for chromosome segregation and transcriptional gene silencing. Heterochromatin protein 1 (HP1) is a conserved chromosomal protein that plays an important role in heterochromatin assembly. We have previously shown that mammalian HP1α and *Schizosaccharomyces pombe* Swi6 are phosphorylated by casein kinase II (CK2) and that this phosphorylation is essential for their function in heterochromatin assembly. In addition to CK2‐mediated phosphorylation, several studies have shown that HP1 proteins undergo additional phosphorylation during mitosis. However, functional significance of the mitotic phosphorylation of HP1 remains unclear. Here, we identified mitotic phosphorylation sites within fission yeast Swi6 and showed that this phosphorylation is involved in chromosome segregation. Using an *Escherichia coli* co‐expression system, we showed that Swi6 is phosphorylated by Ark1, a solo Aurora kinase in *S. pombe*, and mutational analyses revealed that serine residues in the conserved N‐terminal region of Swi6 are the primary targets of Ark1. By expressing mutant Swi6, we confirmed that these serine residues are phosphorylated during mitosis in vivo. Although non‐phosphorylatable or phosphomimic mutations in Swi6 had little effect on heterochromatic silencing, they caused defects in early chromosome segregation and modulated the temperature‐sensitive growth of mutant cells for chromosome passenger complex components. These results suggest that the Ark1‐mediated mitotic phosphorylation of Swi6 is involved in chromosome segregation during mitosis and implicates a conserved regulatory role for the mitotic phosphorylation of HP1 proteins.

## Graphical abstract

Heterochromatin protein 1 (HP1) plays an important role in heterochromatin assembly. HP1 proteins undergo additional phosphorylation during mitosis. However, the functional significance of the mitotic phosphorylation of HP1 remains unclear. We identified mitotic phosphorylation sites within fission yeast Swi6 and showed that this phosphorylation is involved in chromosome segregation and implicates a conserved regulatory role for the mitotic phosphorylation of HP1 proteins.

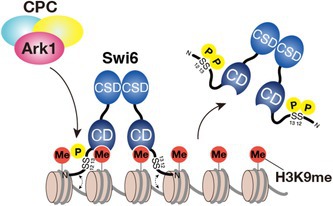

**Abbreviations**
5‐FOA: 5‐fluoroorotic acid
CD: chromodomain
ChIP: chromatin immunoprecipitation
CPC: chromosome passenger complex
CSD: chromoshadow domain

*E. coli*

:
*Escherichia coli*

EDTA: ethylenediaminetetraacetic acid
EMSA: electrophoretic mobility shift assays
EGFP: enhanced green fluorescent protein
H3K9me3: trimethylation of histone H3 lysine 9
HP1: heterochromatin protein 1
PAGE: polyacrylamide gel electrophoresis
qPCR: quantitative PCR
RT‐qPCR: reverse transcription‐quantitative PCR
SAP: shrimp alkaline phosphatase
SDS: sodium dodecyl sulfate
*S. pombe*
: *Schizosaccharomyces pombe*
TBZ: thiabendazole
WT: wild‐type
YEA: yeast extract with adenine

## 1 Introduction

In eukaryotes, genomic DNA in the nucleus is wrapped around histone octamers to form chromatin. Heterochromatin is a highly condensed chromatin structure that plays an important role not only in the regulation of gene expression, but also in the formation of functional domains of chromosomes such as centromeres and telomeres, and in their proper segregation [1]. Post‐translational modifications of histone tails play an important role in the formation of higher‐order chromatin structures. In particular, trimethylation of histone H3 lysine 9 (H3K9me3) is a hallmark of constitutive heterochromatin, and this modification is catalyzed by evolutionarily conserved SUV39H family histone methyltransferases that provide a binding site for heterochromatin protein 1 (HP1) [2, 3, 4]. HP1 family proteins share a basic structure consisting of an amino‐terminal chromodomain (CD) and carboxyl‐terminal chromoshadow domain (CSD) that are linked by an unstructured hinge region. The CD targets H3K9me3, whereas the CSD contributes to dimer formation and the recruitment of various trans‐acting factors [5]. Although the unstructured hinge and N‐ and C‐terminal tails are less conserved, these regions are thought to be involved in isoform‐specific functions. Many eukaryotes express multiple HP1 isoforms; for example, in the fission yeast *Schizosaccharomyces pombe*, two HP1 isoforms, Swi6 and Chp2, have distinct functions in the formation of higher‐order chromatin structures [6, 7, 8].

Similar to histones, the function of HP1 family proteins is regulated by posttranslational modifications; in particular, the phosphorylation of HP1 proteins has been extensively studied in various species [5, 9, 10]. In *Drosophila*, HP1a, an HP1 isoforms, is multiply phosphorylated in tissues, where the phosphorylation levels change dynamically during development [11]. HP1a is primarily phosphorylated by casein kinase II (CK2) at two residues, S15 and S202, located at the N‐ and C‐termini of the protein, and mutations in these residues reduce the heterochromatin‐binding ability of HP1a [12]. In addition to CK2‐mediated phosphorylation, HP1a is phosphorylated at the hinge region, and amino acid substitutions at candidate target residues affect its homodimerization and H3K9me3‐binding ability [13]. Mammalian cells express three HP1 isoforms, HP1α, HP1β, and HP1γ (also known as CBX5, CBX1, and CBX3, respectively). Two‐dimensional electrophoresis analysis revealed that HP1α and HP1γ, but not HP1β, are phosphorylated throughout the cell cycle and hyperphosphorylated during mitosis [14]. We have previously shown that HP1α is multiply phosphorylated by CK2 at N‐terminal serine residues (S11–14), and that this phosphorylation enhances the nucleosome‐binding specificity of HP1α by improving its H3K9me3 recognition via the CD and suppressing its intrinsic DNA binding activity [15, 16, 17]. The N‐terminal phosphorylation of HP1α also contributes to liquid‐droplet formation [18]. In addition to N‐terminal phosphorylation by CK2, HP1α undergoes mitosis‐specific phosphorylation in its hinge region. In human HP1α, S92 is the primary target for phosphorylation, which is catalyzed by Aurora B kinase and dephosphorylated at the end of the M phase [19, 20]. S92‐phosphorylated HP1α preferentially dissociates from chromatin [19], and nonphosphorylatable HP1α (S92A) fails to rescue the mitotic chromosomal instability caused by HP1α depletion [21]. The spatiotemporal dynamics of HP1α have been shown to be essential for accurate chromosome segregation, although a direct relationship with HP1α phosphorylation has not been clearly established [22]. Similar to HP1α, HP1γ has been shown to be phosphorylated in the hinge region (S83) in an M phase‐specific manner, and that this phosphorylation contributes to mitotic chromosome stability [23]. Taken together, these studies suggest the importance that mitotic phosphorylation of HP1 proteins has in ensuring chromosomal stability. However, given that the residues targeted by mitotic Aurora kinases are located in the less conserved hinge region and that not all HP1 isoforms are similarly regulated by mitotic phosphorylation, it remains unclear whether the relationship between M phase‐specific HP1 phosphorylation and mitotic chromosome function is conserved in other eukaryotic species. The fission yeast, *S. pombe*, expresses two HP1 proteins, Swi6 and Chp2, which play distinct roles in heterochromatin assembly. We have previously shown that Swi6 and Chp2 are phosphorylated by CK2 in vivo, and that CK2‐mediated Swi6 phosphorylation is essential for its function in heterochromatin assembly [24]. However, whether these HP1 proteins acquire additional phosphorylation during the M phase remains unclear.

In mammalian cells, Aurora kinase B forms a highly conserved chromosome passenger complex (CPC) with INCENP, survivin, and Borealin/Dasra, which ensures proper chromosome segregation by destabilizing aberrant microtubule attachments and promoting chromosome biorientation on the mitotic spindle [25]. In fission yeast, the Ark1 kinase forms a complex with Pic1/INCENP and Bir1/survivin, and these CPC components play an essential role in chromosome segregation during mitosis as deletion mutants for genes encoding each of them are inviable [26, 27, 28]. A previous report showed that Swi6 contributes to proper localization of the Aurora kinase complex at centromeres, and that when mutations in this complex are combined with *swi6*∆, cells exhibit a marked growth defect by inducing lagging chromosomes at anaphase [29]. However, the molecular details of how Swi6 function is linked to the functions of Ark1 and the CPC remain unclear.

In this study, we characterized the mitotic phosphorylation of *S. pombe* Swi6. We used an *Escherichia coli* co‐expression system to identify the mitotic phosphorylation site(s) in Swi6 and further confirmed its phosphorylation dynamics during mitosis in vivo. Overall, our results indicate that mitotic phosphorylation of Swi6 is involved in chromosome segregation processes and suggest that the relationship between mitotic chromosome stability and M‐phase‐specific phosphorylation of HP1 is evolutionarily conserved.

## 2 Materials and Methods

### 2.1 Strains and Plasmids

The *S. pombe* strains used in this study are described in Table S1, and the media and genetic methods used were previously described [30]. To construct plasmids for producing recombinant Swi6 proteins in *E. coli* cells, the coding sequence of full‐length *swi6* ^+^ or each domain was amplified via PCR and cloned into the pCold I vector (Takara Bio, Shiga, Japan). These plasmids were subjected to site‐directed mutagenesis as previously described [31], using the Pfu Turbo PCR system (Agilent Technologies, Santa Clara, CA, USA). To express Ark1 in *E. coli* cells, the *ark1* ^+^ coding sequence was amplified via PCR and cloned into pACYCDuet‐myc, a pACYCDuet (Merck Millipore, Burlington, MA, USA) derivative containing a sequence for the Myc tag instead of a 6 × His‐tag. To express CK2 in *E. coli* cells, the coding sequences for *cka1* ^+^ and *ckb1* ^+^ were amplified via PCR and cloned into pRSFDuet‐FLAG, a pRSFDuet (Merck Millipore) derivative containing a sequence for the FLAG tag instead of 6 × His‐tag [17]. The primers used for cloning and site‐directed mutagenesis are listed in Table S2.

To express mutant Swi6 proteins from the native *swi6* ^+^ promoter, a pop‐out method was used. The *swi6* ^+^ coding sequence with its potential promoter and terminator regions was first cloned into pBluescript (pAL2pBK), whereafter two restriction enzyme sites (BamHI and PacI sites immediately after the ATG and stop codon, respectively) were introduced through site‐directed mutagenesis (pAL2BP) [6]. The *ura4* ^+^ marker gene was then introduced into the pAL2BP plasmid (pAL2UBP), and using BamHI and PacI sites, the *swi6* ^+^ coding region was replaced with one containing deletions or amino‐acid substitutions. The resultant plasmids were cleaved using MfeI and introduced into the original *swi6* ^+^ locus, and stable transformants were then isolated using a medium lacking uracil. To replace the wild‐type *swi6* ^+^ allele with the mutant *swi6* allele, strains that had lost the *ura4* ^+^ gene through internal homologous recombination were isolated using a counter‐selective medium containing 5‐fluoroorotic acid (5‐FOA). Appropriately replaced clones were verified using the BamHI site that had been introduced immediately after the ATG codon. The same pop‐out method described above was used to obtain strains expressing EGFP fused wild‐type or mutant Swi6 proteins. To simultaneously monitor chromosome behavior during mitosis, strains expressing EGFP‐Swi6 and harboring the *cdc25‐22* allele were transformed with an *hht1*‐*mCherry*‐*kanMX6* fragment. Strains harboring each silencing marker gene (*Kint2::ura4* ^+^ or *otr1R::ade6* ^+^) or temperature‐sensitive allele (*cdc25‐22*, *ark1‐T8‐GFP*, *bir1‐T1*, or *pic1‐T296*) were constructed using standard genetic crosses. The PCR‐based module method was used for the deletion of target genes [32].

### 2.2 Antibodies

The following antibodies were used in this study: anti‐Swi6 (rabbit, home‐made antibodies raised against recombinant Swi6 proteins, diluted to 0.5 μg/mL) [33], anti‐tubulin (T5168, 1:2000; Sigma‐Aldrich, St. Louis, MO, USA), horseradish peroxidase (HRP)‐conjugated anti‐mouse IgG (515–035‐072, 1:1000; Jackson ImmunoResearch, West Grove, PA, USA), and HRP‐conjugated anti‐rabbit IgG (A6667, 1:1000; Sigma‐Aldrich).

### 2.3 Recombinant Protein Production

Recombinant 6 × His‐tagged proteins (wild‐type or mutant Swi6 and its domains) were expressed in BL21(DE3) *E. coli* and purified using immobilized metal affinity chromatography, according to the manufacturer's instructions (TALON; Clontech, Mountain View, CA, USA). Recombinant Swi6 proteins were further purified using anion‐exchange chromatography (SOURCE 15Q; Cytiva, Marlborough, CA, USA). To produce Ark1‐ or CK2‐phosphorylated Swi6 proteins, BL21(DE3) *E. coli* were simultaneously transformed with pCold I‐Swi6 and pACYCDuet‐Ark1 or pRSFDuet‐Cka1/Ckb1, respectively, and selected with ampicillin (for pCold I), chloramphenicol (for pACYCDuet), or Kanamycin (for pRSFDuet). The phosphorylated Swi6 proteins were purified as described above.

### 2.4 Phos‐Tag PAGE and Immunoblotting

The cells were grown in YEA (0.5% yeast extract, 3% glucose, 75 μg/mL adenine) to an absorbance of 1–2 measured at a wavelength of 595 nm. The cells were spun at 4°C and washed once with ice‐cold STOP buffer (150 mM NaCl, 50 mM NaF, 10 mM EDTA, 1 mM NaN_3_, pH 8.0) or distilled water. The cell pellet (2.5 × 10^8^ cells in a 2‐mL screw‐cap tube) was resuspended in 200 μL cell lysis buffer (50 mM HEPES/KOH [pH 7.5], 300 mM potassium acetate, 5 mM magnesium acetate, 20 mM β‐glycerol phosphate, 1 mM EGTA, 1 mM EDTA, 0.1% Nonidet P‐40, 1 mM dithiothreitol, 1 mM phenylmethylsulfonyl fluoride), supplemented with a protease inhibitor mixture (Complete; Roche Applied Science, Basel, Switzerland), and lysed via vortexing with glass beads using a Multi‐Beads Shocker (Yasui Kikai, Osaka, Japan). The cell lysate was diluted with 200 μL cell lysis buffer and spun at 21 880× *g* for 10 min at 4°C. After determining the protein concentration, the cleared lysates were subjected to immunoblotting. Alternatively, whole‐cell lysates were prepared using the alkaline‐TCA method, as previously described [34] with slight modifications. Briefly, the pelleted cells (2 × 10^7^ cells) were resuspended in 500 μL ice‐cold distilled water and mixed with 75 μL Alkali‐2ME solution (1.85 N NaOH, 1.07 M 2‐mercaptoethanol). After the incubation on ice for 5 min, the cell suspension was mixed with 75 μL of 50% TCA solution, further incubated on ice for 5 min, and then spun at 21 880× *g* for 5 min at 4°C. After removing the supernatant, the precipitated proteins were resuspended in 100 μL of SDS‐sample buffer, boiled, and used for immunoblotting. To prepare cell lysates of G2/M‐arrested cells or cells in the M phase, *S. pombe cdc25‐22* mutant strains were used. Cells grown at the permissive temperature (25°C) were collected to prepare cell lysates at the asynchronous state. Cells were synchronized at the G2/M transition through incubation for 4 h at the restrictive temperature (36°C), and a portion of the culture was collected to prepare cell lysates at the G/M‐arrested state. The remaining cells were released to a permissive temperature (25°C), and after 40 min, they were collected to prepare M‐phase cell lysates. For dephosphorylation, the whole‐cell lysate (100 μg) prepared using the former method was incubated with 1 U SAP (Takara Bio) in SAP reaction buffer (50 mM Tris–HCl [pH 9.0], 5 mM MgCl_2_) for 3 h at 37°C. PAGE using a chemical reagent called Phos‐tag (FUJIFILM Wako Pure Chemical Corporation, Osaka, Japan) was performed, as previously described [15, 17, 19]. Briefly, 30 μM phos‐tag‐acrylamide and an equal volume of 10 μM MnCl_2_ were added to a 10% or 12% SDS‐PAGE gel. Prior to protein transfer, the phos‐tag gels were incubated with transfer buffer containing 5 mM EDTA for 20 min and subsequently washed with fresh transfer buffer for 15 min. Proteins were transferred to a polyvinylidene fluoride membrane (Immobilon P; Merck Millipore) using a transfer buffer (25 mM Tris, 190 mM glycine, 20% methanol) and a Mini Trans‐Blot Cell electrophoresis system (Bio‐Rad, Hercules, CA, USA) at 100 V for 1 h. The membranes were washed once with TBST (10 mM Tris–HCl [pH 7.5], 150 mM NaCl, 0.05% Tween20), blocked with blocking buffer (5% [w/v] nonfat dry milk in TBST), and incubated with antibodies diluted in TBST. After washing with TBST, protein signals were detected using chemiluminescence (ECL Western Blotting Detection System, Cytiva) and ChemiDoc Imaging Systems (Bio‐Rad).

### 2.5 Silencing Assay and Reverse Transcription‐PCR Analyses

Silencing assays and reverse‐transcription PCR analyses were performed as described previously [6, 33]. Briefly, cells were grown in YEA medium, and 10‐fold serial dilutions were spotted onto plates containing minimal nonselective medium, minimal medium without uracil (–Ura), or minimal medium containing 5‐FOA. The plates were then incubated at 30°C for 2–4 days.

### 2.6 Chromatin Immunoprecipitation

ChIP analysis was performed as previously described [6, 33]. The primers used for ChIP experiments are listed in Table S2.

### 2.7 Chromatin Fractionation Assays

Chromatin fractionation assays were performed as previously described [6].

### 2.8 Observation of Lagging Chromosomes

To visualize tubulin, cells were transformed with the pREP41‐EGFP‐*atb2* ^+^ plasmid, which was generated by assembling the pREP41‐EGFP plasmid and *atb2* ^+^ coding DNA using the NEBuilder HiFi DNA Assembly kit (NEB, Ipswitch, MA, USA). The *atb2* ^+^ coding DNA was PCR‐amplified using fission yeast genomic DNA as a template. EGFP fluorescence was observed, as previously described [35], with slight modifications. Briefly, cells were grown at 30°C in YEA and harvested for fixation. The cells (1 × 10^7^) were fixed with 100 μL of 100% methanol for 20 s. After fixation, the cells were washed twice with 1× PBS and incubated with 100 μL of 1 ng/mL DAPI with 1× PBS for 5 min. The cells were collected via brief centrifugation and suspended in 50 μL anti‐fade mounting medium (Prolong Diamond, Thermo Fisher Scientific, Waltham, MA, USA). For observation, the cells were placed onto a glass slide and covered with a coverslip. Cell imaging was performed as previously described [36], with slight modifications. Fluorescence images were acquired using SoftWoRx software on a DeltaVision microscope system with an Olympus PlanApo 60× objective lens (Cytiva). A set of Z‐sections was taken at 10 focal planes with 0.3‐μm intervals. The projected images were processed using Fiji software.

### 2.9 Time‐Lapse Observation of Living Cells

Cell imaging was performed, as previously described [36]. To observe mitotic cells, early log‐phase *cdc25‐22* mutant cells were cultured at 36°C for 4 h and then shifted down to 25°C. The cells were placed on a 35‐mm glass‐bottom culture dish (Matsunami, D11130H), which was coated with 0.2% lectin (*w*/vol). Live cell observation was carried out at 25°C. Fluorescence images were acquired using SoftWoRx software on a DeltaVision microscope system with an Olympus PlanApo 60× objective lens (Cytiva). A set of images was obtained every 2 min at 10 focal planes with 0.4 μm intervals to observe EGFP‐Swi6 and Hht1‐mCherry during mitosis. All images were processed using SoftWoRx software (Cytiva). Time‐lapse images were analyzed using Fiji software (https://imagej.net).

### 2.10 Electrophoretic Mobility Shift Assays

EMSAs using DMA probes were performed, as previously described [17, 19].

## 3 Results

### 3.1 *Schizosaccharomyces pombe* Swi6 Is Hyperphosphorylated During Mitosis

**FIGURE 1:**
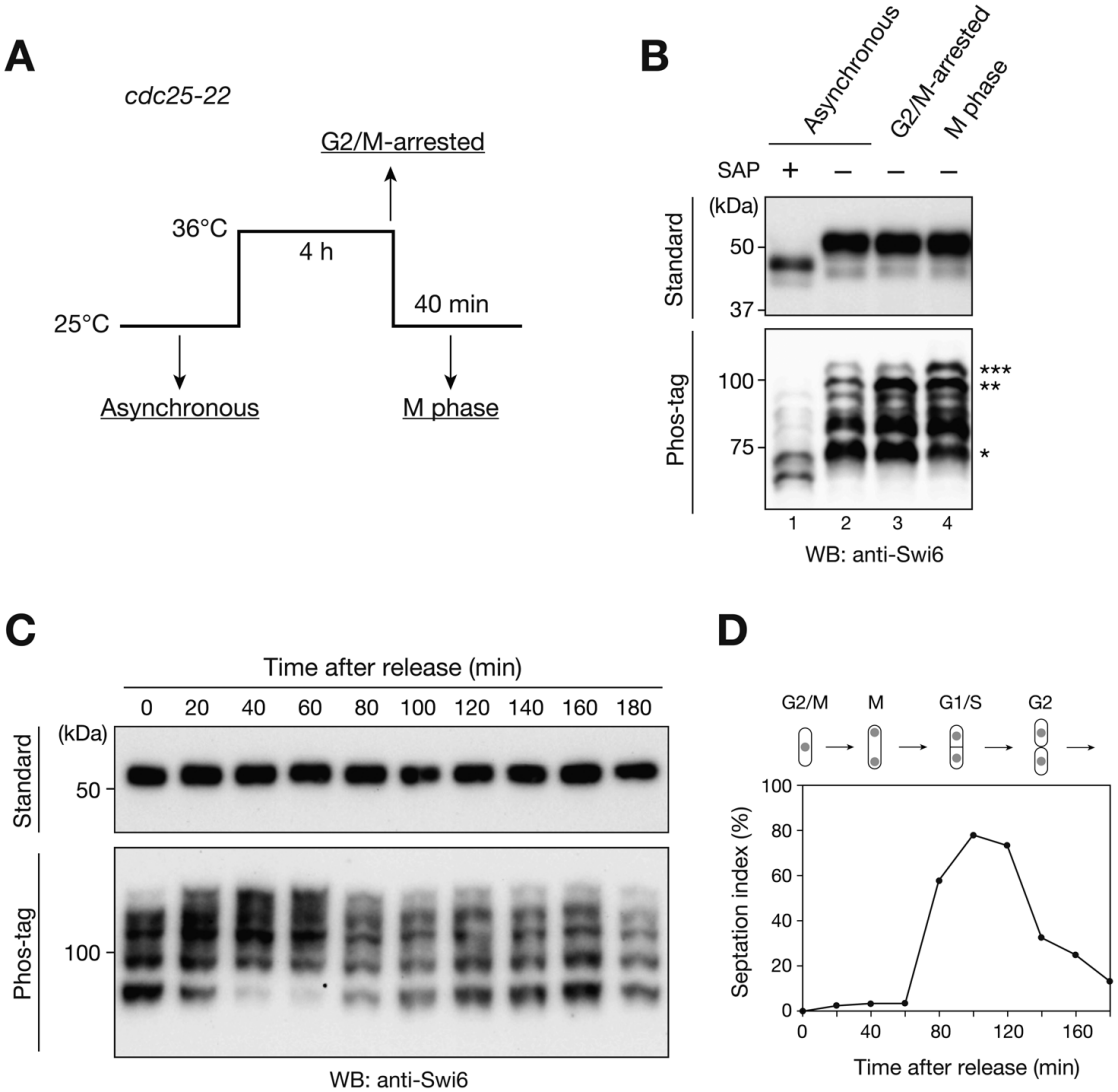
Swi6 is hyperphosphorylated during mitosis. (A) Schematic of the procedure used to prepare cells in the asynchronous state, in the G2/M‐arrested state, or in the M phase using *Schizosaccharomyces pombe cdc25‐22* mutant strains. (B) Phosphorylation patterns of Swi6 in asynchronously growing, G2/M‐arrested, and M‐phase cells. The *cdc25‐22* cells were synchronized at the G2/M transition through incubation for 4 h at the restrictive temperature (36°C) and subsequently released to the permissive temperature (25°C). G2/M‐arrested cells were harvested immediately before release and M‐phase cells harvested 40 min thereafter. Whole‐cell lysates were resolved via standard (top, 10%) or Phos‐tag (bottom, 8%, 30 μM Phos‐tag) sodium dodecyl sulfate‐polyacrylamide gel electrophoresis and analyzed using immunoblotting with anti‐Swi6 antibody. Shrimp alkaline phosphatase (SAP)‐treated samples were used as unphosphorylated (or less phosphorylated) controls. Asterisks indicate the Swi6 species that exhibited an altered pattern during mitosis. (C) Time‐course experiments showing the dynamics of Swi6 phosphorylation. The *cdc25‐22* cells were synchronized at 36°C and released via a temperature shift at 25°C. The cells were harvested every 20 min after release to prepare protein samples, and the Swi6 phosphorylation patterns examined by using standard and Phos‐tag gels as in (B). (D) The septation index is shown along with a schematic illustration of corresponding phases of the cell cycle. The septation peak roughly coincides with the S phase. All experiments were performed at least twice, and representative data are shown.

Swi6 is constitutively phosphorylated by CK2 [24]; however, it is not clear whether phosphorylation occurs at specific stages of the cell cycle, particularly in the M phase. To determine whether Swi6 undergoes M phase‐specific phosphorylation, the temperature‐sensitive *cdc25‐22* allele was used: G2/M‐arrested cells were prepared by culturing cells at a nonpermissive temperature (36°C for 4 h), and M‐phase cells prepared by harvesting cells approximately 40 min after the culture was shifted to a permissive temperature (25°C) (Figure 1A). In the standard sodium dodecyl sulfate‐polyacrylamide gel electrophoresis (SDS‐PAGE) results, the majority of Swi6 prepared from asynchronously cultured cells was detected at the position approximately corresponding to a molecular mass of 50 kDa, and faster migrating bands of ~45 kDa were also detected (Figure 1B, top, lane 2). When the protein samples were treated with shrimp alkaline phosphatase (SAP), both the faster‐ and slower‐migrating bands shifted to much faster bands (Figure 1B, top, lane 1), suggesting that Swi6 is stably phosphorylated throughout the cell cycle and that this phosphorylation somewhat persists after SAP treatment, or that Swi6 experiences post‐translational modification(s) other than phosphorylation.

When the same protein samples were resolved via Phos‐tag PAGE (a chemical that forms a complex with Mn^2+^ ions and selectively captures phosphorylated proteins, thereby slowing their migration during SDS‐PAGE) [37], two major and several minor bands were detected (Figure 1B, bottom, lane 2), indicating that although Swi6 was detected as one major band in the standard SDS‐PAGE gel, it had been multiply phosphorylated with variations. While the band pattern of Swi6 prepared from cells during G2/M arrest or the M phase did not change significantly in the standard SDS‐PAGE gel, the faster‐migrating Swi6 bands in the Phos‐tag gel became weaker (Figure 1B, bottom, lanes 3 and 4, marked with *) and the slower‐migrating bands became stronger (Figure 1B, bottom, lanes 3 and 4, marked with ** and ***) than those detected in the asynchronous sample.

To confirm the dynamics of Swi6 phosphorylation during mitosis, time‐course experiments were performed: *cdc25‐22* cells were harvested every 20 min after release from the restrictive temperature to prepare protein samples, and Swi6 phosphorylation patterns examined using standard and Phos‐tag gels (Figure 1C,D). As shown in the time‐point experiment (Figure 1B), the faster‐migrating Swi6 bands in the Phos‐tag gel became weaker and the slower‐migrating bands became stronger at the M phase (approximately 40–60 min after release), and the phosphorylation patterns were restored during cell cycle progression. Taken together, these results suggest that Swi6 undergoes additional phosphorylation during the M phase.

### 3.2 Determination of the Swi6 Phosphorylation Sites Targeted by Ark1

**FIGURE 2:**
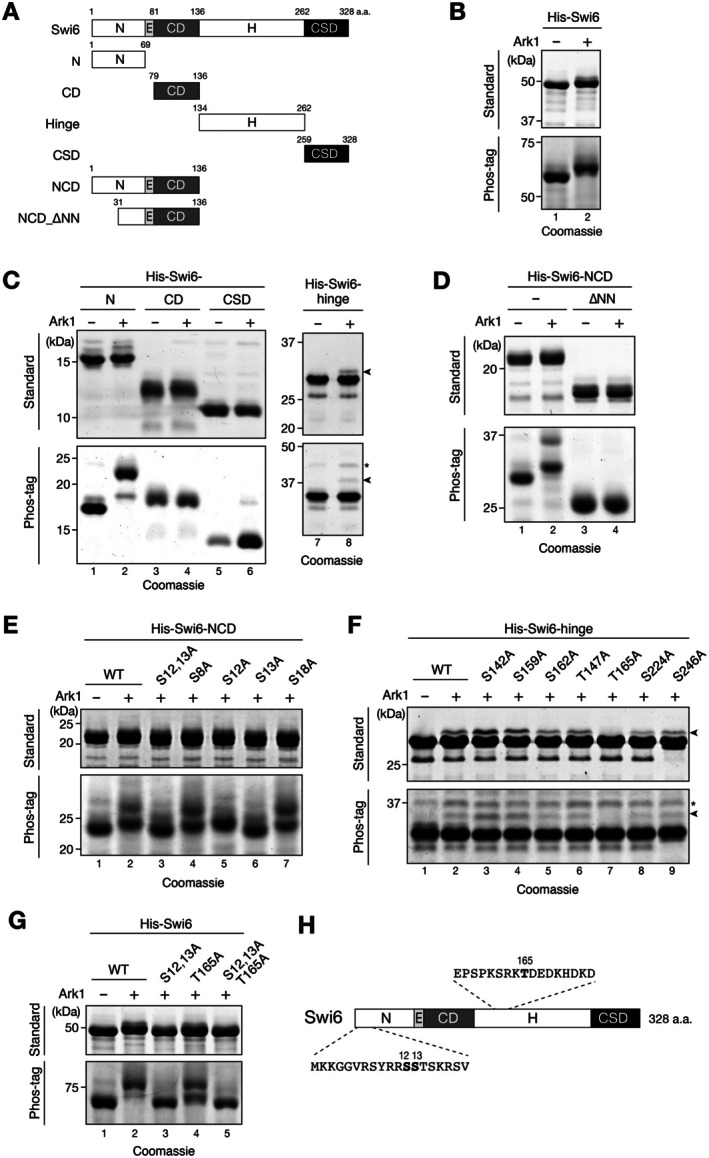
Swi6 is phosphorylated by Aurora kinase. (A) Schematic representation of the full‐length Swi6 and constructs for each of its domains; CD, chromodomain; CSD, chromoshadow domain; E, glutamic acid‐rich region; H, hinge region; N, N‐terminal domain. (B) Swi6 proteins produced in control or Ark1‐co‐expressed *Escherichia coli* cells were purified, resolved through standard or Phos‐tag polyacrylamide gel electrophoresis (30 μM), and visualized via CBB staining. (C) The Swi6 protein was divided into five parts as shown in (A), and each domain (except the E region) was produced in *E. coli* cells with or without Ark1 and analyzed as in (B). The arrowhead indicates peptides corresponding to the H with Ark1‐mediated phosphorylation. The asterisk indicates unrelated peptides that were also detected in the Ark1‐untreated control sample. (D) Recombinant proteins for the Swi6 N‐terminus and CD (Swi6‐NCD), as well as the Swi6‐NCD with a 1–30‐amino acid deletion (Swi6‐NCD_∆NN), were produced in *E. coli* cells with or without Ark1 and analyzed as in (B). (E) The *E. coli* co‐expression assay using a recombinant protein containing the Swi6 N‐terminus and CD (Swi6‐NCD). Wild‐type (WT) Swi6‐NCD and mutants with alanine substitutions at S12, S13, S8, S18, or both S12 and S13 were produced in *E. coli* cells with Ark1 and analyzed as in (B). (F) The *E. coli* co‐expression assay using recombinant protein containing the Swi6 hinge region (Swi6‐hinge). WT Swi6‐hinge and mutants with alanine substitutions at S142, S159, S162, S147, S165, S224 or S246 were produced in *E. coli* cells with Ark1 and analyzed as in (B). The arrowhead indicates peptides corresponding to the H with Ark1‐mediated phosphorylation. The asterisk indicates unrelated peptides that were also detected in the Ark1‐untreated control sample. (G) WT Swi6 and mutants with alanine substitutions at S12 and S13 (S12,13), T165, or S12,13 combined with T165 (S12,13, T165) were produced in *E. coli* cells with Ark1 and analyzed as in (B). (H) Schematic representation of full‐length Swi6 and the residues subject to Ark1‐mediated phosphorylation in the *E. coli* co‐expression system. The mitotically phosphorylated residues are shown in bold letters. All experiments were performed at least twice, and representative results are shown.

In human cells, two Aurora kinases, Aurora A and Aurora B, play important roles in mitotic progression [38], where HP1α is phosphorylated at the hinge region by Aurora B during the M phase [19, 20]. The fission yeast, *S. pombe*, expresses a single Aurora kinase, Ark1 [26], which likely catalyzes the mitotic phosphorylation of Swi6. To determine the candidate of mitotic phosphorylation site(s) in Swi6, we used an *E. coli* co‐expression system that we have previously used to produce human HP1α and Swi6 with CK2‐mediated phosphorylation [15, 17]. When His‐tagged recombinant Swi6 (Figure 2A) was co‐expressed with Ark1, it migrated more slowly in the standard SDS‐PAGE gel than the control Swi6 band did (Figure 2B, top). Delayed migration was clearly visible in the Phos‐tag gel (Figure 2B, bottom), suggesting that Swi6 is stably phosphorylated by Ark1 in the *E. coli* co‐expression system.

To further investigate the Ark1‐mediated phosphorylation site(s) in Swi6, we divided Swi6 into five parts: N‐terminus (N), glutamic acid (E)‐rich region, CD, hinge region (H), and CSD (Figure 2A). Each domain, except the E‐rich region, was then separately expressed with Ark1, and their migration patterns subsequently analyzed. We found that the N‐terminus of Swi6 was efficiently phosphorylated by Ark1 (Figure 2C, lanes 1 and 2), and that a small population of the hinge region was also targeted (Figure 2C, lanes 7 and 8). No clear differences were observed when the CD was coexpressed with Ark1 (Figure 2C, lanes 3 and 4), whereas a very small population of the CSD appeared to be targeted (Figure 2C, lanes 5 and 6).

To determine the phosphorylation site(s) in the N‐terminus of Swi6, we divided the N‐terminus into two parts: the N‐terminal (residues 1–30) and C‐terminal (residues 31–68) half. As the sizes of these peptides are small, we expressed the Swi6 N‐terminus with the E‐rich region and CD (designated as Swi6‐NCD) and examined its phosphorylation states, as well as for Swi6‐NCD wherein the N‐terminal half of the Swi6 N‐terminus (NN) was deleted (Swi6‐NCD_∆NN) (Figure 2A). Swi6‐NCD was efficiently phosphorylated by Ark1, and two slowly migrating bands, major and minor, were detected in the Phos‐tag gel compared with those in the control (Ark1‐untreated Swi6) (Figure 2D, lanes 1 and 2). When Swi6‐NCD_∆NN was expressed with Ark1, no obvious band shift was observed even in the Phos‐tag gel (Figure 2D, lanes 3 and 4), suggesting that Ark1 phosphorylates several residues located in the NN region of the Swi6 N‐terminus.

Four candidate serine residues can be found in the NN region, namely, S8, S12, S13, and S18. To identify the residues targeted by Ark1, we introduced serine‐to‐alanine mutations at the candidate residues found in the NN region of Swi6‐NCD, expressed each mutant with Ark1, and then analyzed their migration patterns (Figure 2E). When the S12A mutation was introduced, the slowly migrating minor band disappeared, but the major band continued to move slower than that of the control (Figure 2E, lane 5). However, introduction of the S13A mutation resulted in the disappearance of the minor band and a shift in migration of the major band to the same position as that of the control (Figure 2E, lane 6). When both the S12A and S13A mutations were introduced, the migration of Swi6‐NCD was almost the same as that when only S13A was introduced (Figure 2E, lane 3). The S8A or S18A mutation did not alter the overall banding pattern (Figure 2E, lanes 4 and 7). These results suggest that S12 and S13 in the Swi6 N‐terminus are phosphorylated by Ark1, and that based on the migration pattern, the major, faster‐migrating band corresponds to Swi6‐NCD phosphorylated at S13 and the minor, slower‐migrating band to Swi6‐NCD phosphorylated at both S12 and S13.

To determine the phosphorylation site(s) in the hinge region, we introduced serine‐to‐alanine mutations at candidate residues in the Swi6 hinge region, expressed each mutant with Ark1, and then analyzed their migration patterns (Figure 2F). When the T165A mutation was introduced, the slowly migrating band disappeared (Figure 2F, lane 7), whereas no detectable change was observed for the other mutations, suggesting that T165 in the hinge region is the primary target of Ark1. To further verify that the three identified residues were phosphorylated by Ark1 in the full‐length Swi6 construct, we introduced a combination of alanine substitutions and examined their effects on Swi6 migration (Figure 2G). The S12A and S13A double mutations were found to abolish most of the Swi6 band shifts observed (Figure 2G, lane 3), whereas the T165A mutation only weakly affected the overall Swi6 band shift (Figure 2G, lane 4). Taken together, these results suggest that S12 and S13 in the N‐terminus of Swi6 are the primary targets of Ark1, and that T165 in the hinge region can also be targeted but with lower efficiency than that of S12 and S13 (Figure 2H). Notably, all three Ark1 target residues (S12, S13, and T165) matched the consensus of Aurora kinase targets ([K/R] × [S/T]) [39]. Although the CSD could be targeted by Ark1 (Figure 2C, lane 6), it was likely not an efficient target based on the results obtained when full‐length Swi6 was tested.

### 3.3 Conserved Serine Residues in the N‐Terminal Region of Swi6 Are Phosphorylated During Mitosis In Vivo

The *E. coli* co‐expression system used in this study revealed that S12 and S13 residues in the N‐terminus of Swi6 are the primary targets of Ark1. Interestingly, the N‐terminus of Swi6 was highly evolutionarily conserved among closely related fission yeast species (Figure S1, highlighted by the yellow box and red rectangle). Although the N‐terminal region of Swi6 was predicted to be unstructured, the degree of conservation was similar to that of the CD or CSD, in marked contrast to the conservation of the hinge region, which was also predicted to be unstructured. In human HP1α, S92 in the hinge region is phosphorylated by Aurora kinase B, and this phosphorylation is important for HP1α function in the M phase [19, 21]. The T165 residue in the Swi6 hinge region was also phosphorylated by Ark1; however, its efficiency was not as high as that of S12 and S13 residues in the *E. coli* co‐expression system, and T165 was not conserved among the closely related fission yeast species (Figure S1, indicated by the red rectangle). In addition, although the amino acid sequence of the CSD is highly conserved among related fission yeast species, no serine or threonine residues matched the consensus sequence of Aurora kinase targets [39] (Figure S1, highlighted by the green box). Based on these observations, we focused on the physiological function of S12 and S13 phosphorylation in the N‐terminus of Swi6.

**FIGURE 3:**
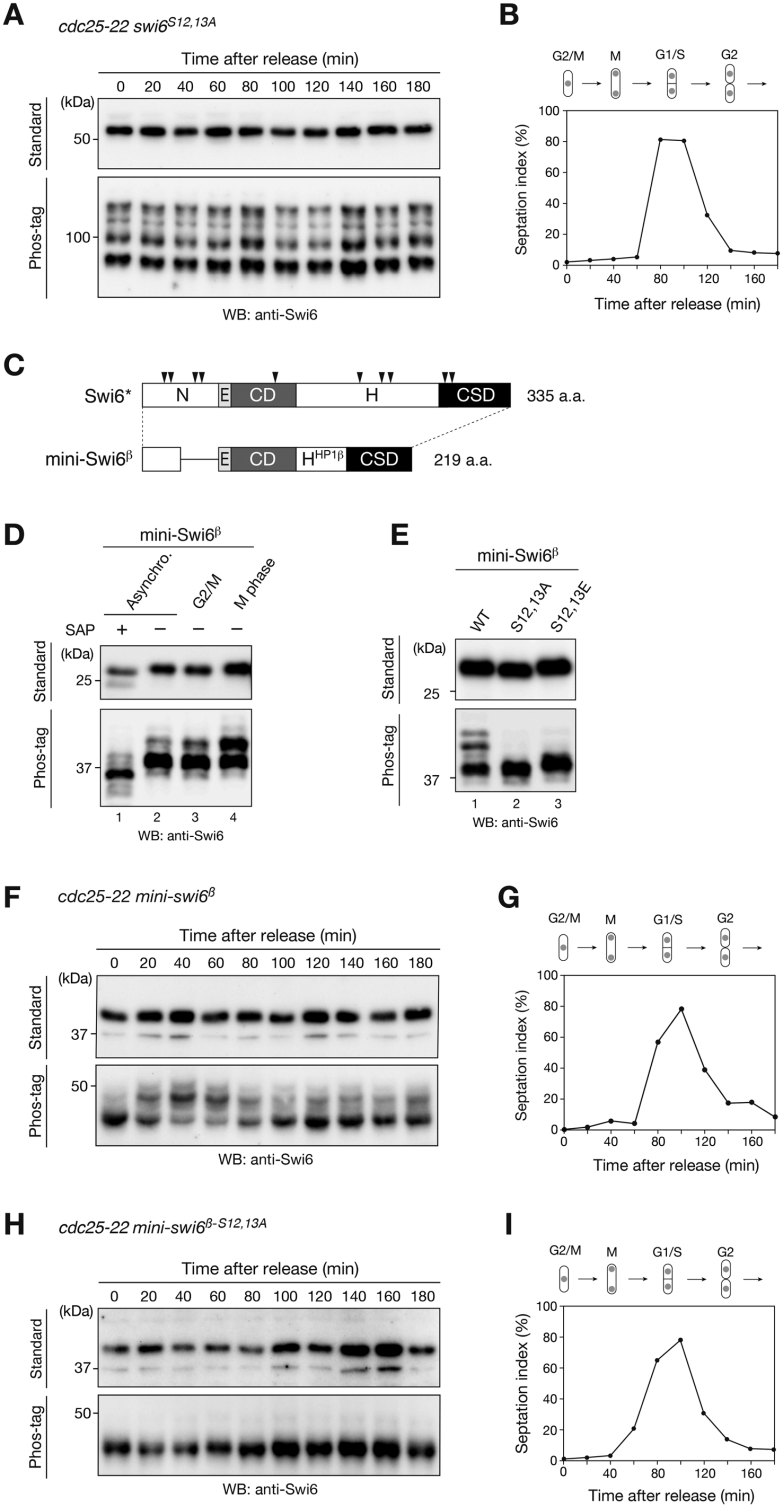
Conserved serine residues in the N‐terminal region of Swi6 are phosphorylated during mitosis in vivo. (A) Time‐course experiments showing the dynamics of the phosphorylation patterns of Swi6^S12,13A^. The *Schizosaccharomyces pombe cdc25‐22* cells expressing Swi6^S12,13A^ were synchronized at the G2/M transition through incubation for 4 h at the restrictive temperature (36°C) and subsequentlyreleased to the permissive temperature (25°C). The cells were harvested every 20 min after release to prepare protein samples, and the phosphorylation patterns examined by using standard and Phos‐tag gels. Whole cell lysates were resolved using standard (top, 10%) or Phos‐tag (bottom, 8%, 30 μM Phos‐tag) sodium dodecyl sulfate‐polyacrylamide gel electrophoresis and analyzed via immunoblotting with anti‐Swi6 antibody. (B) The septation index of c*dc25‐22 swi6* ^*S12,13A*^ cells is shown along with a schematic illustration of corresponding phases of the cell cycle. (C) Schematic representation of full‐length Swi6 and the chimeric Swi6 containing the hinge region of human HP1β (mini‐swi6β). The positions of casein kinase II‐phosphorylatable serine residues are indicated by the black arrowheads. (D) Phosphorylation patterns of mini‐Swi6β in asynchronously growing, G2‐arrested, and M‐phase cells. The *cdc25‐22* cells expressing mini‐Swi6β were analyzed. (E) Immunoblotting analysis of cells expressing mini‐Swi6β with amino acid substitutions. (F) Time‐course experiments showing the dynamics of miniSwi6β phosphorylation. The *cdc25‐22* cells expressing miniSwi6β were synchronized at 36°C and released via a temperature shift at 25°C. The cells were harvested every 20 min after release to prepare protein samples, and the miniSwi6β phosphorylation patterns examined by using standard and Phos‐tag gels as in (B). (G) The septation index of c*dc25‐22 mini‐swi6β* cells is shown along with a schematic illustration of corresponding phases of the cell cycle. (H) Time‐course experiments showing the dynamics of miniSwi6β phosphorylation. The *cdc25‐22* cells expressing miniSwi6β^S12,13A^ were synchronized at 36°C and released via a temperature shift at 25°C. The cells were harvested every 20 min after release to prepare protein samples, and the miniSwi6β^S12,13A^ phosphorylation patterns examined by using standard and Phos‐tag gels as in (B). (I) The septation index of c*dc25‐22 mini‐swi6β* ^*S12,13A*^ cells is shown along with a schematic illustration of corresponding phases of the cell cycle. All experiments were performed at least twice, and representative results are shown.

To investigate whether S12 and S13 residues in the N‐terminus of Swi6 are phosphorylated by Ark1 in vivo, *S. pombe cdc25‐22* cells expressing mutant Swi6 containing the amino acid substitutions S12A and S13A (Swi6^S12,13A^) from the endogenous *swi6* locus were generated. Time‐course experiments were performed to analyze the dynamics of Swi6^S12,13A^ phosphorylation during mitosis using standard and Phos‐tag gels (Figure 3A,B). Although the overall phosphorylation pattern of Swi6^S12,13A^ was similar to that of the wild‐type at the beginning of the time‐course experiments (compare the band pattern of Swi6 in Figure 1C, 0 min and in Figure 3A, 0 min), the patterns did not change markedly during cell cycle progression, which was stark contrast to the pattern of Swi6^WT^ (compare the band pattern of Swi6^WT^ in Figure 1C, 40–60 min and Swi6^S12,13A^ in Figure 3A, 40–60 min). These results suggest that Swi6 is phosphorylated at S12 and S13 during the M phase.

Although changes in the migration pattern of mutant Swi6 suggest that the S12 and S13 residues of Swi6 are phosphorylated during the M phase in vivo, the constitutive phosphorylation of Swi6, presumably by CK2, makes it difficult to evaluate its mitotic phosphorylation. A previous study showed that Swi6 is phosphorylated by CK2 at several residues in the N‐terminus and hinge region [24]. To further investigate the mitotic phosphorylation of Swi6, we expressed several Swi6 mutants predicted to exhibit reduced CK2‐mediated phosphorylation and analyzed their migration patterns. Initially, we deleted approximately half of the distal region of the Swi6 N‐terminus (residues 35–65) (Figure S2A) and confirmed that the resulting mutant, Swi6^∆35–65^, was expressed and retained its silencing function comparable with that of control Swi6 (Figure S2B [lane 4] and Figure S2C). Next, we replaced the hinge region with that of human HP1β to reduce the number of residues potentially phosphorylated by CK2 (Figures 3C and S2A) and confirmed that the resulting mutant Swi6, termed mini‐Swi6β, was expressed and also retained its silencing function comparable with that of control Swi6 and Swi6^∆35–65^ (Figure S2B, lane 5 and Figure S2C). In addition, as observed for wild‐type Swi6, EGFP‐fused mini‐Swi6β localized several nuclear spots corresponding to heterochromatic regions in a *clr4* ^+^ dependent manner (Figure S2D). The migration pattern of mini‐Swi6β became much simpler than that of wild‐type Swi6; one major and two slowly migrating minor bands were detected in the Phos‐tag gel for the sample prepared from cells in the asynchronous condition (Figure 2D, lane 2 and Figure 2E, lane1), and the intensity of the minor bands became stronger during the M phase (Figure 2D, lane 4), suggesting that these bands correspond to mini‐Swi6β that has undergone mitotic phosphorylation.

To confirm the dynamics of mini‐Swi6β phosphorylation during mitosis, *S. pombe cdc25‐22* cells expressing mini‐Swi6β were generated, and time‐course experiments performed using standard and Phos‐tag gels (Figure 3F,G). As shown in the time‐course experiment for wild‐type Swi6 (Figure 1C,D), the faster‐migrating mini‐Swi6β bands detected in the Phos‐tag gel became weaker and the slower‐migrating bands became stronger at the M phase (approximately 40–60 min after release), and the phosphorylation patterns were restored during cell cycle progression (Figure 3F,G). Importantly, the introduction of amino acid substitutions at S12 and S13 in mini‐Swi6β (S12,13A or S12,13E) resulted in the loss of these slowly migrating minor bands (Figure 2E, lanes 2 and 3), and time‐course experiments confirmed that the migration pattern of mini‐Swi6β^S12,13A^ did not change markedly during cell cycle progression (Figure 3H,I). Taken together, these results strengthen our conclusion that Swi6 is phosphorylated at S12 and S13 during the M phase.

### 3.4 Nonphosphorylatable Mutations in Swi6 Weakly Enhance Heterochromatin Silencing

**FIGURE 4:**
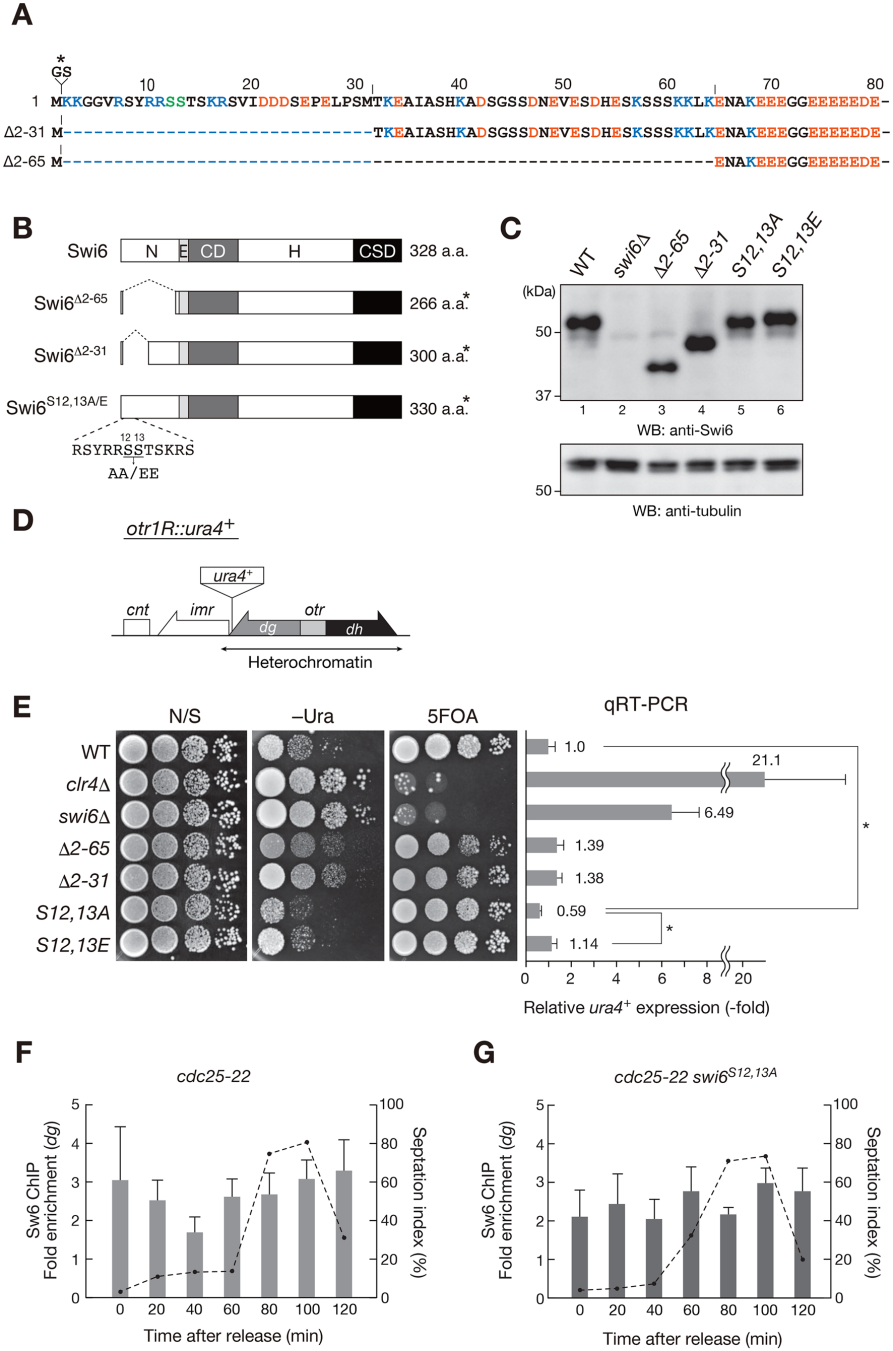
Unphosphorylatable Swi6 mutant enhances heterochromatin silencing. (A) Amino acid sequence of the Swi6 N‐terminal region (1–80 amino acids). Amino acids are shown, with acidic and basic amino acids indicated in orange and blue, respectively. Serine residues that could be phosphorylated by Ark1 and casein kinase II are shown in green. To replace the wild‐type *swi6* ^+^ allele with the mutant *swi6* allele, a BamHI site was introduced immediately after the ATG codon; the position of two additional amino acids, glycine and serine, is indicated by an asterisk (*). (B) Schematic diagram of full‐length Swi6 and Swi6 mutants with N‐terminal deletion (∆2–65 or ∆2–31) or amino acid substitutions (S12,13A or S12,13E). (C) Immunoblotting analysis of cells expressing wild‐type (WT) or mutant Swi6 proteins. Immunoblotting with an anti‐α‐tubulin antibody is shown as a loading control. (D) Diagram of the right side of *centromere 1* (right) in *Schizosaccharomyces pombe*. The position of the silencing reporter gene, *otr1R::Ura4* ^+^, is shown. (E) Heterochromatic silencing assays of WT and mutant *swi6* strains. The silencing status at the centromeric *otr1R::Ura4* ^+^ was evaluated. Tenfold serial dilutions of the indicated strains were spotted onto nonselective medium (N/S), minimal medium without uracil (–Ura), and minimal medium containing 5‐fluoroorotic acid (5‐FOA) (left). Expression levels of the *ura4* ^+^ silencing reporter were evaluated via quantitative reverse‐transcription PCR (qRT‐PCR) analyses (right). Means and standard deviations of at least three independent experiments are shown. Statistical significance was determined using ANOVA. *p < 0.05. (F and G) Time‐course chromatin immunoprecipitation (ChIP) analysis of Swi6 (F) or Swi6^S12,13A^ (G) associated with pericentromeric *dg* regions, relative to *act1* ^+^. Immunoprecipitated DNA was subjected to quantitative PCR analysis. Means and standard deviations of at least three independent experiments are shown.

Phosphorylation of HP1 proteins, particularly M phase‐specific phosphorylation, is thought to be involved in chromosome segregation; however its role in gene silencing is not well understood. The N‐terminal region of Swi6, which is phosphorylated specifically in the M phase is thought to be unstructured, yet it is as well conserved as the CD and CSD are, which form stable 3D structures, suggesting that this region may be involved in Swi6 functions beyond the M phase. To investigate the potential role of the conserved N‐terminal region and residues undergoing mitotic phosphorylation, we expressed mutant Swi6 containing nonphosphorylatable mutations (S12,13A), phosphomimic mutations (S12,13E), or N‐terminal deletion (∆2–65 or ∆2–31) (Figure 4A–C) and then examined their function to silence marker genes inserted into the pericentromeric region of *centromere 1* (*otr1R::ura4* ^+^) (Figure 4D).

Deletion of the Swi6 N‐terminus, either ∆2–65 or ∆2–31, resulted in a weak silencing defect for *otr1R::ura4* ^+^, although the degree of silencing derepression was milder than that of *clr4*∆ or *swi6*∆, suggesting that the unstructured N‐terminal region partially contributes to the silencing function of Swi6 (Figure 4E). This result is consistent with a previous observation that N‐terminal‐deleted Swi6 (∆1–73) showed a mild defect in iodine staining, indicating a lower efficiency of mating‐type switching [40]. Introduction of the nonphosphorylatable mutations, S12,13A or the phosphomimic mutations, S12,13E, had little effect on gene silencing, suggesting that the phosphorylation of S12 and S13 residues contributes little to the silencing function of Swi6 (Figure 4E). These results were consistent with the localization analysis showing that the EGFP‐fused Swi6^S12,13A^ and Swi6^S12,13E^ mutants formed clear nuclear spots in interphase cells, similar to the wild‐type Swi6 (Figure S3). Interestingly, the introduction of the nonphosphorylatable mutations, S12,13A, appeared to slightly enhance silencing, as the cells expressing Swi6S^12,13A^ grew poorly on the –Ura plate and the expression level of *otr1R::ura4* ^+^ evaluated by reverse‐transcription PCR analyses was lower than that of wild‐type cells (Figure 4E). The mechanism underlying the enhanced silencing is unknown; however, it is possible that the M phase‐specific phosphorylation of Swi6 affects its dynamics throughout the cell cycle, which may have altered its silencing function. Although it remains unclear how the evolutionarily conserved N‐terminus contributes to Swi6 function, alanine substitution of the target serine residues may have altered its function by reducing regional hydrophilicity.

### 3.5 N‐Terminal Phosphorylation of Swi6 Regulates Its Chromatin Association During Mitosis

As mitotically phosphorylated human HP1α is readily dissociates from chromatin [19], the Swi6 chromatin‐binding ability may likely be similarly regulated by mitotic phosphorylation. While performing chromatin fractionation assays and testing whether the Swi6 chromatin‐binding ability is altered by mitotic phosphorylation, we found that the mitotic phosphorylation of mini‐Swi6β was lost during the preparation of mitotic cell lysates, presumably due to the phosphatase activity in the prepared lysates, making it difficult to evaluate the role that mitotic phosphorylation plays in modulating the Swi6 chromatin binding ability (Figure S4). To investigate whether mitotic phosphorylation of Swi6 is involved in its chromatin‐binding ability, time‐course chromatin immunoprecipitation (ChIP) assays were performed: *cdc25‐22* cells were harvested every 20 min after release from restrictive temperature conditions, fixed, and then subjected to ChIP assays using an anti‐Swi6 antibody (Figure 4F). As previously reported [41], the levels of Swi6 in the pericentromeric *dg* regions (Figure 4D) gradually decreased after release until 40 min and gradually recovered during cell cycle progression (Figure 4F). Importantly, a similar decrease in Swi6 levels was not observed in *cdc25‐22* cells expressing Swi6^S12,13E^ (Figure 4G). These results suggest that N‐terminal phosphorylation of Swi6 contributes to its partial dissociation from pericentromeric regions, as previously demonstrated for human HP1α.

### 3.6 Expression of Swi6 Mutant Defective in N‐Terminal Phosphorylation Results in Chromosome Segregation Defects

**FIGURE 5:**
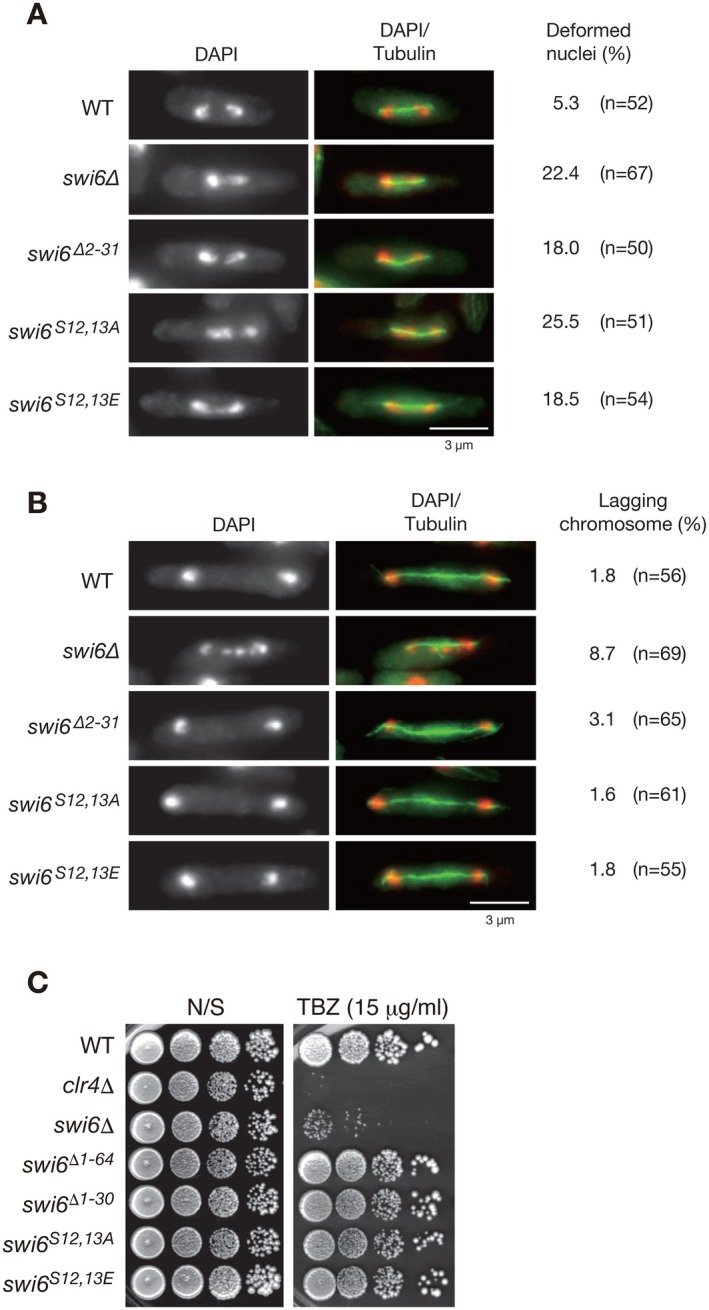
Expression of Swi6 mutant defective in N‐terminal phosphorylation results in chromosome segregation defects at metaphase/early anaphase but not at late anaphase. (A and B) Analysis of chromosome segregation defects and lagging chromosomes in the *swi6* mutants. EGFP‐tubulin (green); DNA was stained with DAPI (red). Scale bar, 3 μm. Chromosome segregation defects at metaphase/early anaphase (A) and the lagging chromosome frequency at late anaphase (B) in the mutants. The number of cells with deformed nuclei (A) or lagging chromosomes (B) is shown on the right of each representative image along with the number of cells examined. (C) Spotting assay to examine thiabendazole (TBZ) sensitivity for cells expressing wild‐type (WT) or each of the mutant Swi6 proteins. The *clr4*∆ cells were used as a control.

The formation of centromeric heterochromatin is critical for proper kinetochore function, and Swi6 has been shown to be involved in accurate chromosome segregation during mitosis [42, 43]. To investigate whether N‐terminal phosphorylation of Swi6 is involved in chromosome stability during mitotic progression, enhanced green fluorescent protein (EGFP)‐fused α‐2‐tubulin was expressed in wild‐type or *swi6*∆ cells, cells expressing mutant Swi6 with S12,13A or S12,13E mutations, or those lacking N‐terminal regions (∆2–64 or ∆2–30), and the behavior of the mitotic chromosome was monitored under a fluorescence microscope (Figure 5A,B). Consistent with previous studies, *swi6*∆ cells showed higher frequencies of nuclear deformation in metaphase/early anaphase (Figure 5A) and lagging chromosomes in late anaphase (Figure 5B). Although the frequencies were lower than those of *swi6*∆ cells, cells expressing Swi6^∆2–31^ also showed higher frequencies of both nuclear deformation and lagging chromosomes, suggesting that the N‐terminal region of Swi6 plays a role in accurate chromosome segregation. Interestingly, cells expressing mutant Swi6 defective in N‐terminal phosphorylation (Swi6^S12,13A^ or Swi6^S12,13E^) also showed a higher frequency of nuclear deformation in metaphase/early anaphase; however, the rate of lagging chromosomes in late anaphase was comparable with that of wild‐type cells, suggesting that Swi6 N‐terminal phosphorylation specifically contributes to the chromosome segregation process at metaphase/early anaphase.

To investigate the role of N‐terminal phosphorylation of Swi6 in chromosome segregation, we analyzed the localization of EGFP‐fused wild‐type and mutant Swi6 containing S12,13A or S12,13E amino acid substitutions in living cells. We monitored the behavior of the chromosomes by expressing H3 fused with mCherry. During this analysis, we observed that bright Swi6 spots, corresponding to clustered centromeres, were separating at the onset of prometaphase (Figure S5A–C, indicated by white arrowheads). We determined the onset of anaphase by observing the separation of chromosomes labeled by H3‐mCherry (Figure S5A–C, indicated by yellow arrowheads). In cells expressing either Swi6^S12,13A^ or Swi6S^12,13E^, the time from prometaphase to anaphase was longer than in cells expressing wild‐type Swi6 (Figure S5D). These results suggest that N‐terminal phosphorylation of Swi6 plays a critical role in the smooth transition from prometaphase to anaphase. Since both the nonphosphorylatable (S12,13A) and phosphomimic (S12,13E) mutations increased the length of time from prometaphase to anaphase, the phosphorylation and dephosphorylation cycle may be important for proper Swi6 function in mitosis.

To investigate the role that Swi6 N‐terminal phosphorylation plays during mitosis, we exposed these mutant cells to thiabendazole (TBZ), a drug that destabilizes microtubules and induces chromosomal loss during late anaphase. The control strains, *clr4*∆ and *swi6*∆, showed hypersensitivity to TBZ (Figure 5C). However, strains expressing mutant Swi6 with nonphosphorylatable mutations (S12,13A), phosphomimic mutations (S12,13E), or N‐terminal deletions (∆2–65 or ∆2–31) showed minor sensitivity to TBZ (Figure 5C). This result is consistent with the microscopic observations (Figure 5A,B) and suggests that the N‐terminal phosphorylation of Swi6 plays a minor role in late anaphase.

### 3.7 N‐Terminal Phosphorylation of Swi6 Is Functionally Linked to the Chromosome Passenger Complex

In fission yeast, Ark1 kinase forms a complex with Pic1/INCENP and Bir1/survivin, and these CPC components play an essential role in chromosome segregation during mitosis; deletion mutants for genes encoding each of these components are inviable [26, 27, 28]. A previous report showed that Swi6 contributes to proper localization of the Aurora kinase complex at centromeres, and that when mutations in this complex are combined with *swi6Δ*, cells exhibit a marked growth defect by inducing lagging chromosomes at anaphase [29]. Based on the relationship between Swi6 and the Aurora kinase complex, we investigated whether amino‐acid substitutions at S12 and S13 in Swi6 modulate growth defects caused by mutations in the Aurora kinase complex.

**FIGURE 6:**
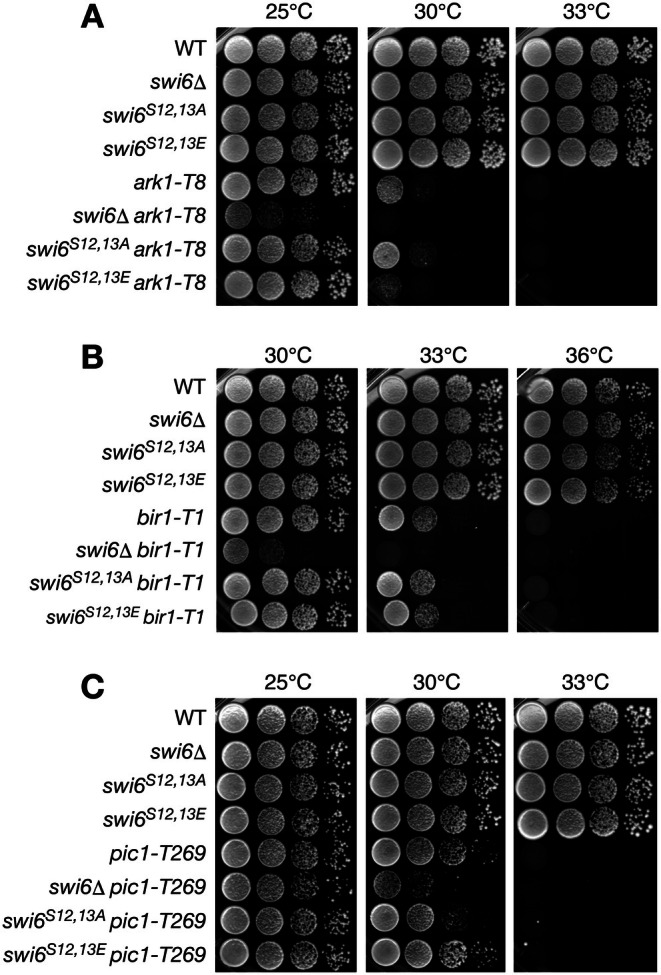
Swi6 N‐terminal phosphorylation is functionally linked to the chromosome passenger complex. (A–C) Serial dilutions of the indicated strains were spotted onto YEA plates and incubated at the indicated temperatures.

The *swi6*∆ strain or strains expressing mutant Swi6^S12,13A^ or Swi6^S12,13E^ grew normally at three different temperatures (25°C, 30°C, and 33°C) (Figure 6A–C). As previously reported, the *ark1‐T8*, *bir1‐T1*, and *pic1‐T269* temperature‐sensitive mutant strains grew normally at the permissive temperature (25°C), grew slowly at the semi‐permissive temperature (30°C), and failed to grow at the restrictive temperature (33°C) [29] (Figure 6A–C). When the temperature‐sensitive alleles, *ark1‐T8* and *bir1‐T1*, were combined with *swi6*∆, the strains showed a severe growth defect, even at the nonpermissive temperature (25°C) (Figure 6A,B). A similar synergistic effect with *swi6*∆ was observed for the *pic1‐T269* allele at the semi‐permissive temperature (30°C) (Figure 6C). When the *ark1‐T8* allele was combined with the *swi6* ^*S12,13A*^ allele, temperature‐sensitive growth was weakly restored, whereas when combined with the *swi6* ^*S12,13E*^ allele, temperature‐sensitive growth was enhanced (Figure 6A, 30°C), suggesting that mitotic phosphorylation of Swi6 affects Ark1 function. In contrast, when the *pic1‐T269* allele was combined with the *swi6* ^*S12,13A*^ mutant allele, temperature‐sensitive growth was slightly enhanced, whereas when combined with the *swi6* ^*S12,13E*^ mutant allele, temperature‐sensitive growth was restored (Figure 6C, 30°C), suggesting that mitotic phosphorylation of Swi6 is functionally linked to Pic1. No apparent change was observed when the *bir1‐T1* allele was combined with either the *swi6* ^*S12,13A*^ or *swi6* ^*S12,13E*^ allele (Figure 6B, 33°C). Taken together, these results suggest that the mitotic phosphorylation and/or dephosphorylation states of Swi6 are involved in the proper functioning of the CPC complex.

### 3.8 Ark1‐Mediated Phosphorylation of Swi6 Reduces Its DNA‐Binding Activity

In a previous study, we showed that mitotic phosphorylation suppresses the DNA‐binding ability of human HP1α, and that mitotically phosphorylated HP1α preferentially dissociates from chromatin [19]. In the M phase, HP1α is phosphorylated at S92 in the hinge region. As S92 is surrounded by clusters of basic amino acid residues important for DNA binding [17, 19], it is likely that the negative charge of phosphorylated S92 inhibits the DNA binding ability of HP1α through the hinge region. Considering that the N‐terminal region of Swi6, which undergoes mitotic phosphorylation, also contains scattered basic amino acid residues (Figure S1, highlighted in blue), M phase‐specific phosphorylation may regulate the DNA‐binding ability of Swi6 as it does in HP1α.

**FIGURE 7:**
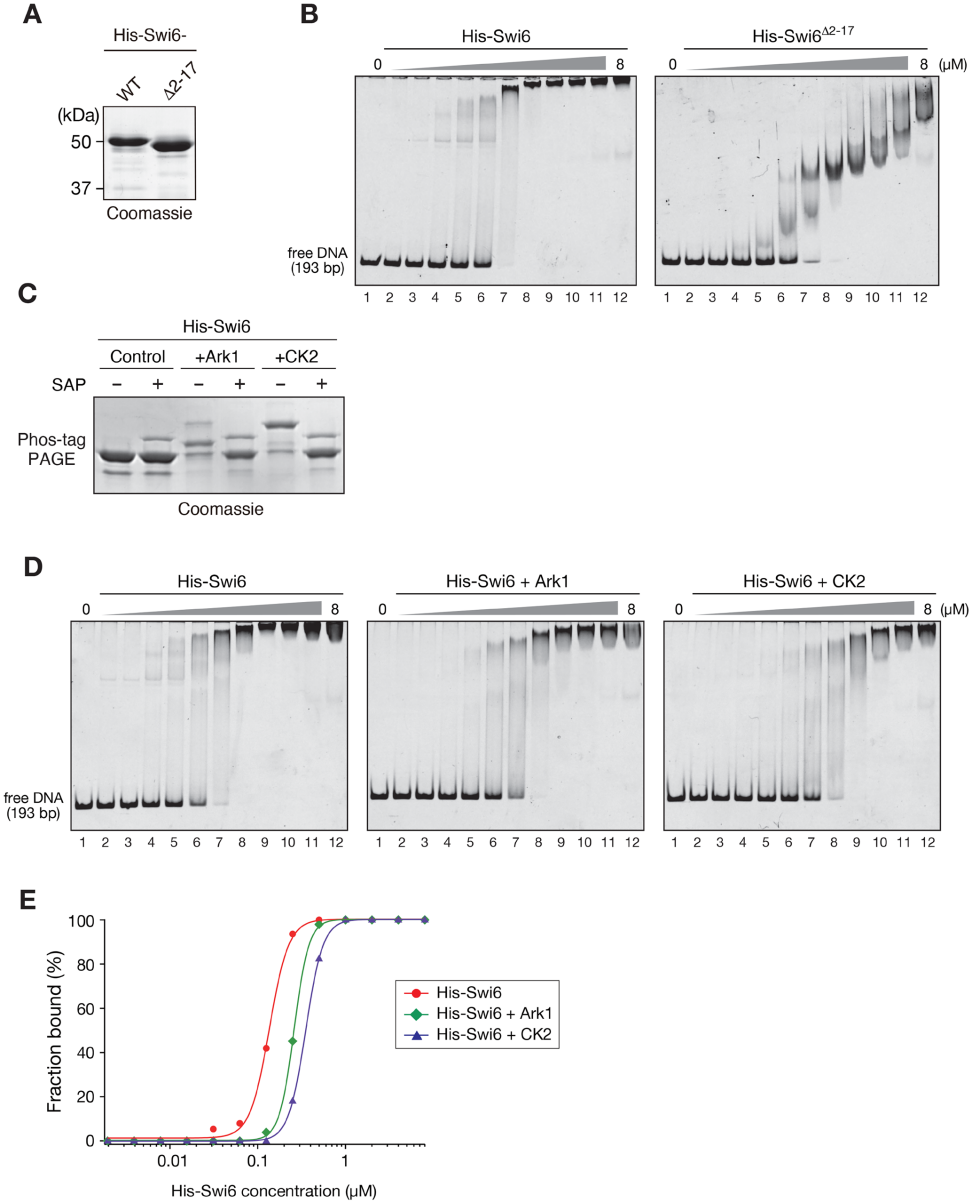
Ark1‐mediated phosphorylation of Swi6 reduces its DNA‐binding activity. (A) Wild‐type (WT) and mutant Swi6 (Swi6^∆2–17^) proteins were purified, resolved using standard polyacrylamide gel electrophoresis (PAGE), and visualized by CBB staining. (B) Representative results of the electrophoretic mobility shift assays (EMSAs) performed with the WT and mutant Swi6. Different concentrations of the Swi6, from 0 to 20 μM (0.6‐fold dilutions), were incubated with 193‐bp 601 DNA. (C) Control and Ark1‐ or casein kinase II (CK2)‐phosphorylated Swi6 proteins were purified, separated with standard or Phos‐tag PAGE, and visualized via CBB staining. Shrimp alkaline phosphatase (SAP)‐treated samples were used as unphosphorylated (or less phosphorylated) controls. (D) Representative results of the EMSAs performed with control and Ark1‐ or CK2‐phosphorylated Swi6. (E) Quantification of the EMSAs performed with control and Ark1‐ or CK2‐phosphorylated Swi6. The bound DNA fractions were estimated from the intensity of the unbound DNA (1‐unbound fraction), and plotted against the Swi6 concentration. All EMSA experiments were repeated at least twice and representative results are shown.

To investigate whether the N‐terminal region of Swi6 is involved in its DNA‐binding ability, we prepared full‐length Swi6 and Swi6 lacking the N‐terminus (Swi6^∆2–17^) as recombinant proteins (Figure 7A) and examined their DNA‐binding ability through electrophoretic mobility shift assays (EMSAs) (Figure 7B). Deletion of the N‐terminus significantly reduced the ability of Swi6 to bind DNA and altered the pattern of shifted DNA probes (Figure 7B), suggesting that, similar to human HP1α, both the N‐terminus and hinge region of Swi6 contribute to its DNA‐binding ability. To investigate the effect that mitotic phosphorylation of Swi6 has on its DNA‐binding ability, we produced a recombinant Swi6 protein with mitotic phosphorylation using an *E. coli* co‐expression system (Figure 7C) and examined its DNA‐binding ability via EMSA (Figure 7D,E). As a control, we also produced Swi6 with CK2‐mediated phosphorylation and examined its DNA‐binding ability. As previously shown [17], CK2‐mediated phosphorylation reduced the ability of Swi6 to bind DNA. Although the effect was not as evident as that of CK2 phosphorylation, Ark1‐mediated mitotic phosphorylation suppressed Swi6 DNA‐binding (Figure 7D,E). While the amino acid substitution from serine to glutamic acid is often used to mimic phosphorylated serine, the recombinant Swi6 protein containing S12,13E showed similar DNA‐binding activity as that of wild‐type Swi6 (Figure S6), suggesting that Ark1‐mediated phosphorylation may have a stronger effect on its DNA‐binding activity. Taken together, these results indicate that the N‐terminus of Swi6 contributes to DNA‐ or nucleosome‐binding, and that its binding mode is modulated by mitotic phosphorylation.

## 4 Discussion

In this study, we focused on Swi6, an HP1 proteins found in fission yeast, and found that it undergoes M phase‐specific phosphorylation. Using an *E. coli* co‐expression system, we showed that S12 and S13 residues in the N‐terminal region of Swi6 are the major phosphorylation sites of Ark1, an Aurora kinase found in fission yeast (Figure 2), and that these sites are indeed phosphorylated during the M phase in vivo (Figure 3). Amino acid substitution experiments revealed that mitotic phosphorylation had little effect on the silencing function of Swi6 (Figure 4) but was involved in metaphase/early anaphase chromosome segregation (Figure 5) and CPC function (Figure 6). Furthermore, we showed that an evolutionarily conserved N‐terminal region of Swi6 contributes to its DNA‐binding ability, and that M‐phase phosphorylation mediated by Ark1 alters this (Figure 7).

The substrates of Aurora kinases have been extensively studied in various species [39, 44]. For fission yeast Swi6, several residues, including S12 and S13, have been reported to be phosphorylated [39, 45, 46, 47, 48, 49]; however, the phosphorylation of S12 and S13 residues has not been detected in a phosphoproteomic analysis of Aurora kinase targets or during the mitotic cell cycle [39, 46]. Although trypsin or other lysyl endopeptidases are routinely used to digest proteins so as to detect phosphorylated peptides, when lysine‐ or arginine‐rich sequences are in the vicinity of residues undergoing phosphorylation, the fragment size of the digested peptides tends to be shortened, which may hinder the detection of phosphorylated peptides. This may explain why S12 and S13 residues could not be represented as major mitotic phosphorylation sites in previous studies [39, 46]. Although phosphoproteomic analysis is a powerful method for identifying the targets of specific kinases, the combination of phosphoproteomics with the strategy used in this study, such as an *E. coli* co‐expression system and verification through mutational analysis, appears to be effective for identifying the phosphorylation sites of specific kinases.

Although the mitotic phosphorylation of HP1 proteins has been studied in human HP1α and HP1γ, its evolutionary conservation, particularly in relation to M‐phase progression and chromosome segregation, is not well understood. The finding that Ark1 phosphorylates specific residues in the conserved N‐terminal region of Swi6 may indicate that the role of mitotic phosphorylation of HP1 proteins is conserved across species. In addition, while the role that phosphorylation of the hinge region plays in HP1 function has been extensively studied (human HP1α and HP1γ undergo mitotic phosphorylation in their hinge region), the mitotic function of other HP1 proteins may also be regulated by phosphorylation in regions other than the hinge region. Interestingly, both human HP1α and fission yeast Swi6 can bind DNA through clusters of basic amino acid residues in the N‐terminal and hinge regions [5, 17, 19], and mitotic phosphorylation occurs in proximity to these clusters. The chromatin‐binding ability of HP1 proteins could likely be achieved through the combinatorial action of the N‐terminus and hinge region, which could be attenuated through phosphorylation at either of these regions, thereby regulating the mitotic function of HP1.

As mitotically phosphorylated human HP1α readily dissociates from chromatin [19], the chromatin‐binding ability of Swi6 could likely be similarly regulated by mitotic phosphorylation. However, we could not directly show that Swi6 chromatin‐binding was altered by mitotic phosphorylation, because the mitotic phosphorylation of Swi6 was lost during the preparation of mitotic cell lysates (Figure S4). However, time‐course ChIP analysis revealed that Swi6 levels in pericentromeric *dg* regions decreased during the M phase (Figure 4F) and that a similar decrease was not observed for Swi6^S12,13A^ (Figure 4G), suggesting that Swi6 N‐terminal phosphorylation also contributes to the transient dissociation of Swi6 from chromatin during mitosis, as demonstrated for human HP1α.

Our microscopic observation revealed that cells expressing Swi6^S12,13A^ or Swi6^S12,13E^ showed defects in chromosome segregation at metaphase/early anaphase, but not at late anaphase (Figures 5A,B and S5). This is consistent with the sensitivity of these mutant cells to TBZ. In a previous study, defects in chromosome segregation during mitosis were classified into six classes [50], and the defects associated with cells expressing mutant Swi6 were similar to those found in class 1 (chromosome segregation was delayed from metaphase to early anaphase, but the delay caught up by late anaphase) or class 3 (chromosome segregation was delayed until late anaphase, but the chromosomes were segregated as the spindle elongates). The phenotypes observed for cells expressing mutant Swi6 suggest delayed migration of the kinetochore to the spindle pole body. The mutant Swi6 protein was not phosphorylated by Ark1 and retained its DNA‐binding activity in the pericentromeric region, which may inhibit proper kinetochore formation during chromosome segregation.

How HP1 regulates CPC function throughout the cell cycle is not fully understood. In human cells, HP1 interacts with CPC components through the CDS and recruits the CPC to centromeres during the late S phase [25, 51, 52]. Aurora B activity is regulated in several steps: Aurora B activation requires binding to INCENP and is coupled to CPC localization [25]. As cells enter mitosis, Aurora B phosphorylates histone H3 serine 10 (H3S10); phosphorylation of H3S10 promotes the dissociation of HP1 from centromeric heterochromatin with H3K9me3 [53, 54]. HP1 has also been shown to bind to the CPC and enhance the enzymatic activity of Aurora kinase B in CPC [20, 25]. Whether a similar regulatory mechanism is evolutionarily conserved and occurs in fission yeast is unknown. However, the fact that mutations in the N‐terminal region of Swi6 altered the growth of temperature‐sensitive mutants of *ark1* and *pic1* at the restrictive temperature suggests that the phosphorylation and/or dephosphorylation states of Swi6 may be involved in Pic1/INCENP‐mediated CPC recruitment and/or activation of Ark1.

In this study, we demonstrated that the expression of Swi6^S12,13E^, which mimics phosphorylation, exhibits an opposite effect on the temperature‐sensitive growth of ark1‐T8 and pic1‐T269 mutants (Figure 6). However, the underlying molecular mechanism remains unclear. As shown in human cells [55], Pic1 likely binds to both Ark1 and Swi6, promoting Ark1‐mediated Swi6 phosphorylation. Swi6 phosphorylation may then facilitate the release of Pic1 from chromosomes. The combination of *ark1‐T8* and Swi6^S12,13E^ may cause chromosome segregation defects by promoting premature Pic1 (INCENP) dissociation from chromosomes. In *pic1‐T269* mutant cells, inefficient Ark1 phosphorylation of Swi6 could cause chromosome segregation defects due to prolonged Pic1 retention on chromosomes, which could be alleviated by Swi6^S12,13E^ expression. Although previous studies have shown genetic interactions between Swi6 and CPC components [29], their physical interactions have not been demonstrated in fission yeast. Further studies using antibodies that specifically recognize phosphorylated Swi6 could help improve our understanding of the link between mitotic Swi6 phosphorylation and CPC function.

## Author Contributions

A. Hayashi, H. Tagami, M. Oki, and J.‐i. Nakayama conceived and designed the research. Y. Yoshimura, M. Tanaka, M. Suzuki‐Matsubara, A. Hayashi, R. Nakagawa, G. Nishibuchi, and J.‐i. Nakayama performed the experiments. A. Hayashi and J.‐i. Nakayama wrote the manuscript with the input from all the authors.

## Conflicts of Interest

The authors declare no conflicts of interest.

## Supporting information


**Figures S1‐S6:** fsb271190‐sup‐0001‐Figures.pdf.

**Tables S1 and S2:** fsb271190‐sup‐0002‐Tables.pdf.

## Data Availability

The authors declare that the data supporting the findings of this study are available within the paper and its Supporting Information file.
