# Supplementary material for: Mitotic Phosphorylation of Swi6/HP1 Regulates Its Chromatin Binding and Chromosome Segregation
Source: FASEB J. 2025 Nov 2;39(21):e71190. doi: 10.1096/fj.202500384R (PMC12580653; doi:10.1096/fj.202500384R)
Supplement: Supplementary file 1 — Figures S1‐S6: fsb271190‐sup‐0001‐Figures.pdf. [file FSB2-39-e71190-s002.pdf]

```
S. cryophilus  -MVKNVRSYRRSSTSKRSVVDEDSEPELPNMTKEIPQTRASDGSISDDEPLIKHEKLEKK 59
S. octsporus   -MVKNVRSYRRSSTTKRSVLDEDSEPELPSMSKEALEANNVDNISSEDEPLVKHEKPEKN 59
S. pombe       MKKGGVRSYRRSSTSKRSVIDDDSEPELPSMTKEAI-ASHKADSGS-----SDNEVE-SD 53
                    ********* **** * ******* * **           *        *

                                                        Chromo domain
S. cryophilus  AEK---GNIADDATGKNADEELNNDEEEEESEEEYVVEKVLKHRLARKGGGYEYLLKWEG 116
S. octsporus   TGKETIGDDDAAGKNTNEETDQDNGEEDDDAEEEYVVEKVLKHRLARRGGGYEYLLKWEG 119
S. pombe       HES---KSSSKKLKENA--KEEEGGEEEEEDE--YVVEKVLKHRMARKGGGYEYLLKWEG 106
                                        **    *  ********** ** ************

S. cryophilus  YDDPSDNTWSREADCDGCKDLVEAYWEERGGRP---DA-SKRK---------RPSRARKP 163
S. octsporus   YDDPSDNTWSTEADCDGCKDLVVAYWEERGGRP---DATSKRK---------RLGRGRKP 167
S. pombe       YDDPSDNTWSSEADCSGCKQLIEAYWNEHGGRPEPSKRKRTARPKKPEAKEPSPKSRKTD 166
               ********** **** *** *  *** * ****

S. cryophilus  ESKEPAVKSQKI-SKSEATKNTRHSEEEEEEEEEEKHELSSPIKAPSPEKETPQKKEVTPQ 222
S. octsporus   ENKETSVKS----RRVSDPEPARPSVEPEEEHEERKEVSSPTKTPSPENHTAEKRESPRK 223
S. pombe       EDKHDKDSNEKIEDVNEKTIKFADK---------SQEEFNENGPPS--GQPNGHIESDNE 215
               * *                                 *       **         *

S. cryophilus  DGHVEPKENVQSVKELSPSPSPIPKKEKQSGASPKKAHLK--VPTLPDEKELTGQQVEKY 280
S. octsporus   NGHAEPKENVPSPN----GPSSLPRKEELPSASPKKNHLK--TPALPEMKELTAQQVERY 277
S. pombe       SKSPSQKESNESED---------IQIAETPSNVTPKKKPSPEVPKLPDNRELTVKQVENY 266
                     **   *                       *       * **   ***  *** *

                                  Chromoshadow domain
S. cryophilus  DMWEDLVASIDTIERKDDGTLEIYLTWKNGAVSHYPSTITNKKCPQKMLQFYESHLTFRE 340
S. octsporus   DTWEELVASIDTIERKDDGTLEIYLTWKNGAVSHYPSSITNKKCPQKMLQFYESHLTFRE 337
S. pombe       DSWEDLVSSIDTIERKDDGTLEIYLTWKNGAISHHPSTITNKKCPQKMLQFYESHLTFRE 326
               * ** ** *********************** ** ** **********************

S. cryophilus  NE 342
S. octsporus   NE 339
S. pombe       NE 328
               **
```

Supplementary Figure S1. N-terminal region of Swi6 is highly conserved among three fission yeast species

Amino acid sequence alignment of Swi6 in three closely related fission yeast species: *Schizosaccharomyces cryophilus*, *S. octosporus*, and *S. pombe*. The N-terminal region of Swi6 conserved in three fission yeast species, the chromodomain, and the chromoshadow domain are indicated by yellow, blue, and green boxes, respectively. The positions of mitotically phosphorylated residues are indicated by a red box. Residues conserved in three fission yeast species are indicated by asterisks below, and acidic and basic amino acid residues in the N-terminal region marked in red and blue, respectively.

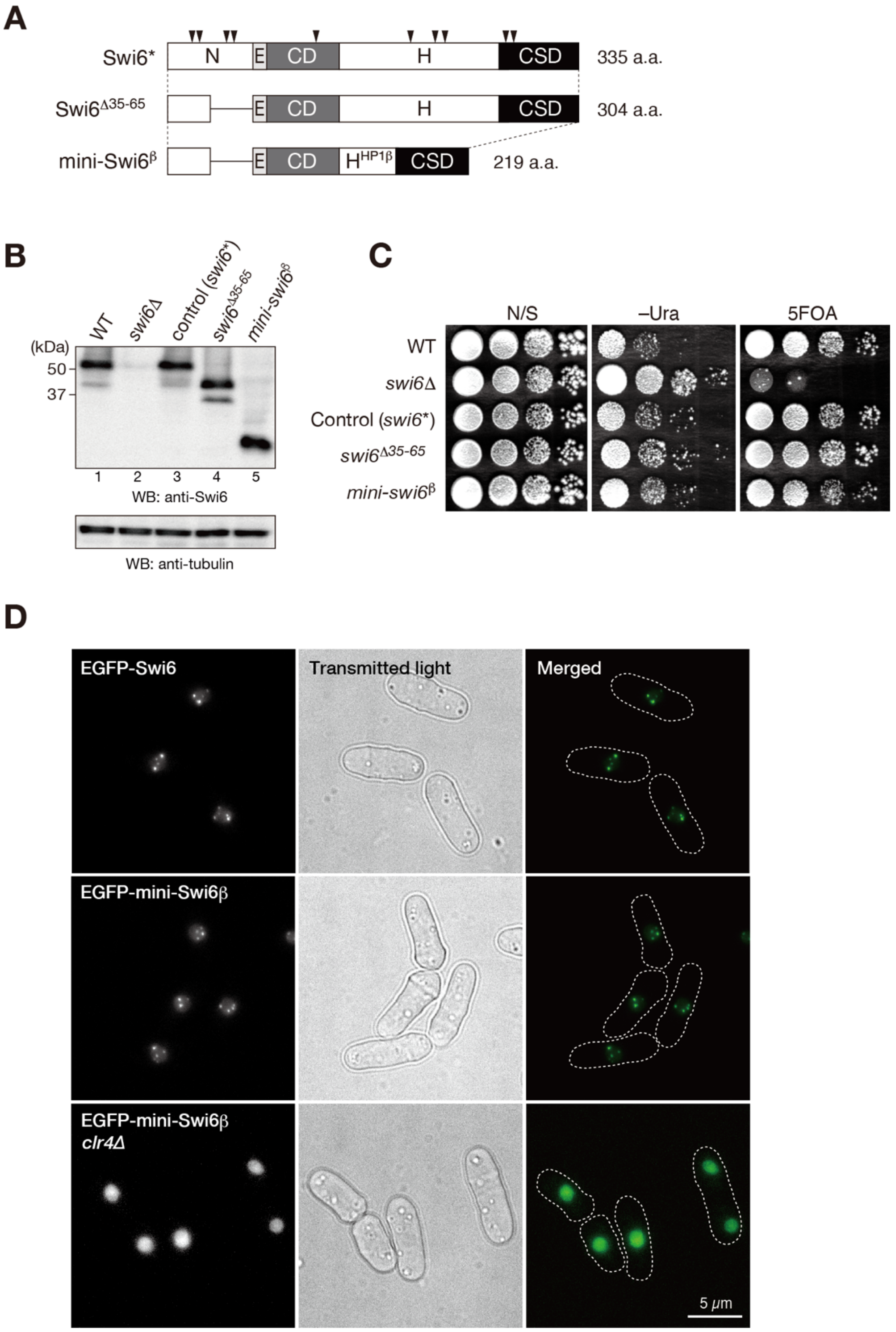
A
Swi6*
N
E
CD
H
CSD
335 a.a.
Swi6Δ35-65
304 a.a.
mini-Swi6β
HHP1β
219 a.a.
B
WT
swi6Δ
control (swi6*)
swi6Δ35-65
mini-swi6β
(kDa)
50
37
1 2 3 4 5
WB: anti-Swi6
WB: anti-tubulin
C
N/S
–Ura
5FOA
WT
swi6Δ
Control (swi6*)
swi6Δ35-65
mini-swi6β
D
EGFP-Swi6
Transmitted light
Merged
EGFP-mini-Swi6β
EGFP-mini-Swi6β
clr4Δ
5 μm

Supplementary Figure S2. Conserved serine residues in the N-terminal region of Swi6 are phosphorylated during mitosis in vivo

(**A**) Schematic representation of full-length Swi6, Swi6 mutant with an N-terminal deletion (Δ35–65), and chimeric Swi6 with the hinge region of human HP1β (mini-swi6β). The positions of casein kinase II-phosphorylatable serine residues are indicated by black arrowheads. (**B**) Immunoblotting analysis of expressed Swi6 proteins. Immunoblotting with an anti-β-tubulin antibody is shown as a loading control. (**C**) Silencing assay for centromeric heterochromatin (*otr1R::ura4*$^{+}$). A tenfold serially diluted culture of the indicated strain was spotted onto nonselective medium (N/S), minimal medium without uracil (–Ura), or minimal medium containing 5-fluoroorotic acid (5-FOA) to evaluate *ura4*$^{+}$ expression. (**D**) Localization of EGFP-fused wild-type Swi6 (EGFP-Swi6) and EGFP-fused mini-Swi6β (EGFP-mini-Swi6β). EGFP-mini-Swi6β formed several nuclear spots similar to those of EGFP-Swi6. In *clr4*Δ cells, the EGFP-mini-Swi6β spots were dispersed within the nucleus. Scale bar, 5 μm.

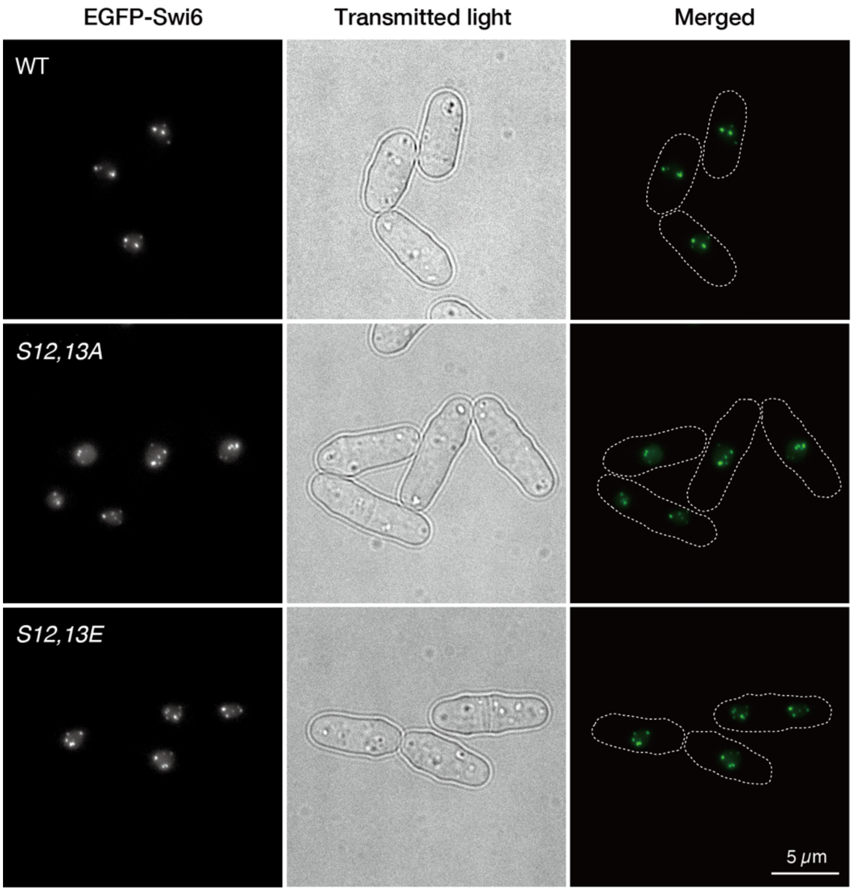

Supplementary Figure S3. Localization of wild-type and mutant Swi6 in asynchronous cells

Wild-type (WT) Swi6 fused with EGFP (EGFP-Swi6) formed several nuclear spots in asynchronously growing cells. EGFP-Swi6 with amino acid substitutions at positions 12 and 13 (S12,13A or S12,13E) formed similar nuclear spots to those of WT EGFP-Swi6. Scale bar, 5 μm.

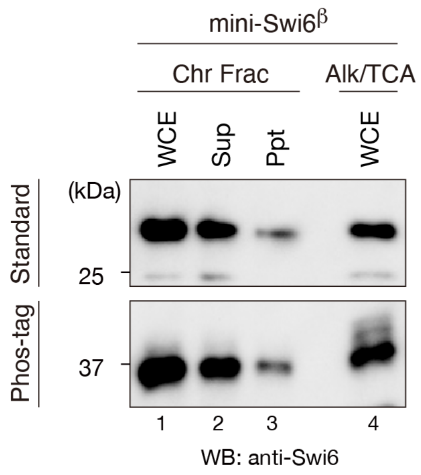

Supplementary Figure S4. Chromatin fractionation assays of mini-Swi6β

Chromatin fractionation assays were preformed using asynchronously growing cells that expressed mini-Swi6β. Whole cell extracts (WCEs) were prepared for the chromatin fractionation assays. The soluble (Sup) and insoluble, chromatin-enriched (Ppt) fractions derived from the WCEs were resolved using standard or Phos-tag polyacrylamide gel electrophoresis (30 μM) and analyzed via immunoblotting with an anti-Swi6 antibody. WCEs prepared using the alkaline-TCA method (Alk/TCA, see Materials and Methods) were used as a control to detect the phosphorylated form of mini-Swi6β.

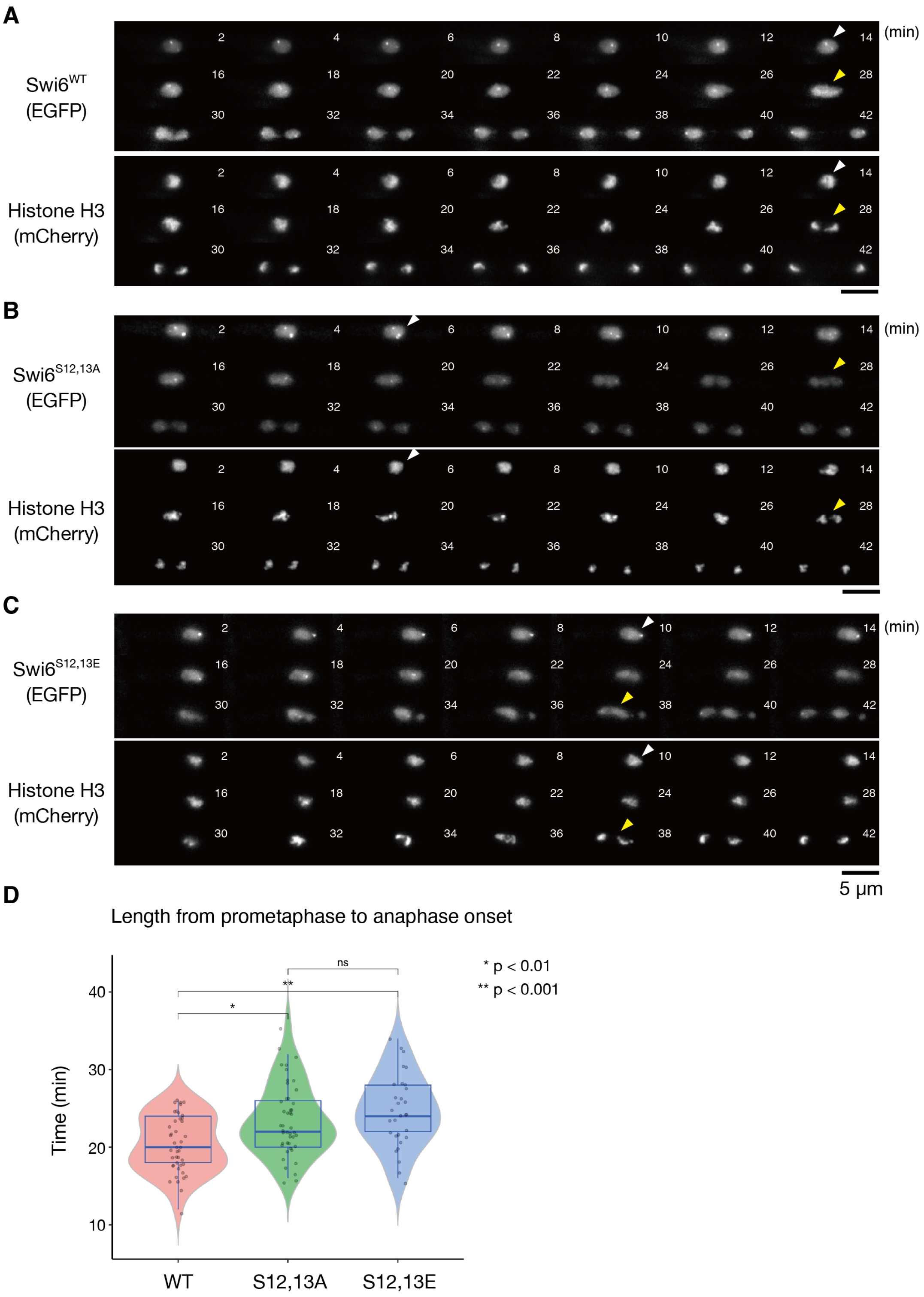
A
Swi6WT
(EGFP)
Histone H3
(mCherry)
(min)
2 4 6 8 10 12 14
16 18 20 22 24 26 28
30 32 34 36 38 40 42
B
Swi6S12,13A
(EGFP)
Histone H3
(mCherry)
C
Swi6S12,13E
(EGFP)
Histone H3
(mCherry)
5 μm
D
Length from prometaphase to anaphase onset
Time (min)
10
20
30
40
WT
S12,13A
S12,13E
ns
*
**
* p < 0.01
** p < 0.001

Supplementary Figure S5. Localization of EGFP-Swi6 during the transition from prometaphase to anaphase

(**A–C**) The localization of the EGFP-fused wild-type (A), S12,13A (B), and S12,13E (C) mutants of Swi6 was analyzed in living cells. Chromosome behavior was also analyzed using the simultaneously expressed Hht1-mCherry. Images were taken every two minutes during mitosis. White arrowheads indicate the onset of prometaphase, which is defined as the separation of the EGFP-Swi6 focus. The bright EGFP-Swi6 foci, localized at the peri-centromeres, are divided with the SPB at the onset of prometaphase. Yellow arrowheads indicate the onset of anaphase, defined as the separation of chromosomes. Scale bar, 5 μm. (**D**) Length from prometaphase to anaphase of cells expressing EGFP-fused wild-type or mutant Swi6. One-way ANOVA test was performed using R (R version 4.4.2). * $p < 0.01$, ** $p < 0.001$.

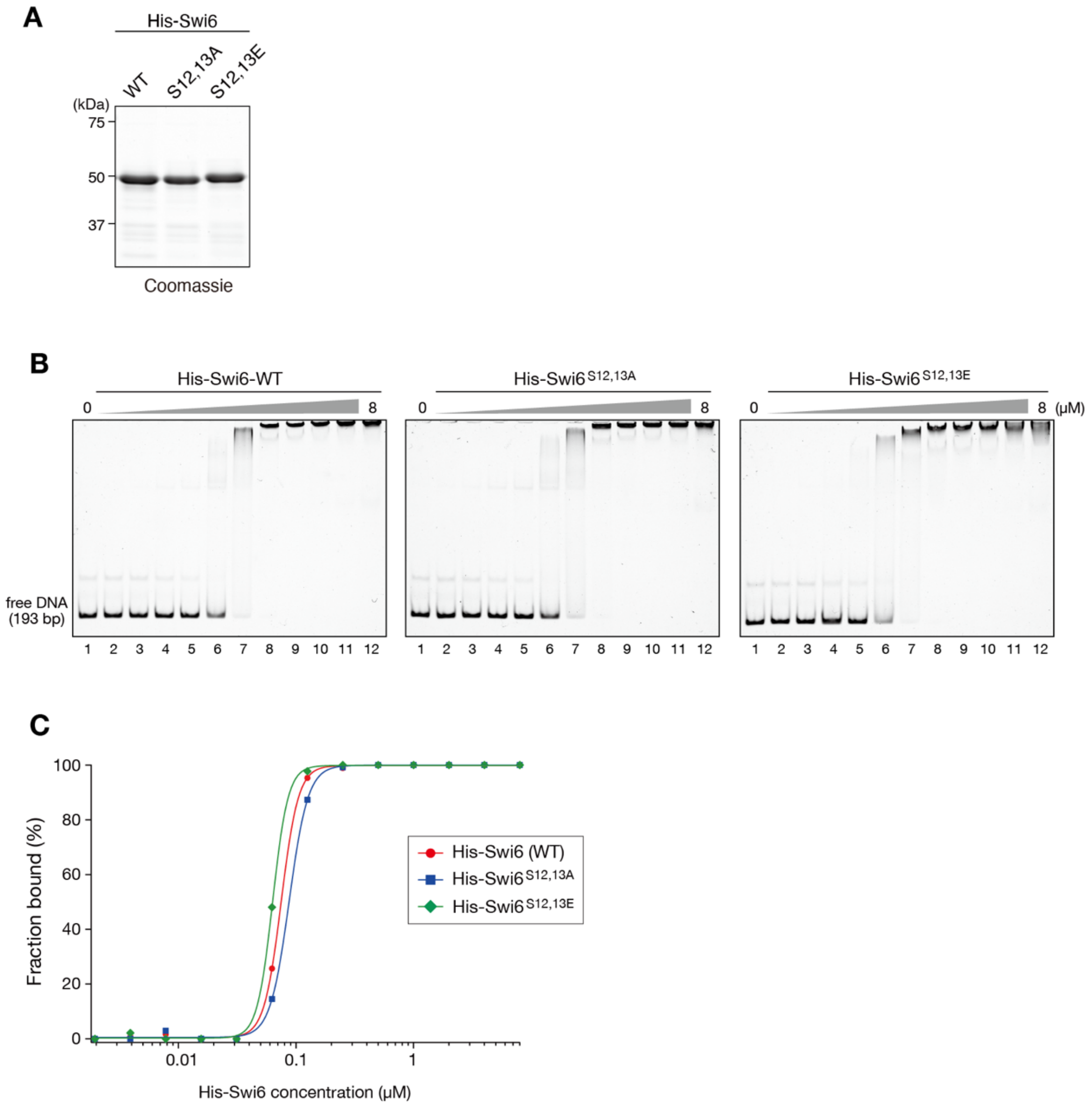

Supplementary Figure S6. Non-phosphorylatable or phosphomimetic amino acid substitutions have little effect on Swi6 DNA binding activity

(**A**) His-tagged, wild-type (WT), and mutant Swi6 (Swi6^{S12,13A} or Swi6^{S12,13E}) proteins were purified, resolved using standard polyacrylamide gel electrophoresis, and visualized via CBB staining. (**B**) Representative results of the electrophoretic mobility shift assays (EMSAs) performed with WT and mutant Swi6. Different concentrations of the Swi6, from 0–8 μM (0.6-fold dilutions), were incubated with 193-bp 601 DNA. (**C**) Quantification of the EMSAs performed with WT and mutant Swi6. The bound DNA fractions were estimated from the intensity of the unbound DNA (1-unbound fraction) and plotted against the Swi6 concentration. All EMSA experiments were repeated at least twice, and representative results are shown.
