# Supplementary material for: Mitotic Phosphorylation of Swi6/HP1 Regulates Its Chromatin Binding and Chromosome Segregation
Source: FASEB J. 2025 Nov 2;39(21):e71190. doi: 10.1096/fj.202500384R (PMC12580653; doi:10.1096/fj.202500384R)
Supplement: Supplementary file 2 — Tables S1 and S2: fsb271190‐sup‐0002‐Tables.pdf. [file FSB2-39-e71190-s001.pdf]

Supplementary Table S1. List of *S. pombe* strains used in this study

Name	Genotype	Source
HKV067	h^- *leu1-32 ura4-D18 ade6-216 cdc25-22*	from H Kato
SPM4058	h^- *leu1-32 ura4-D18 ade6-216 cdc25-22 swi6::swi6-S12,13A*	This study
FY648	h^+ *leu1-32 ura4-DS/E ade6-210 otr1R::*$ura4^+$	Allshire (1994)
SPIT235	h^+ *leu1-32 ura4-DS/E ade6-210 otr1R::*$ura4^+$ *clr4Δ::kanMX6*	Ishida (2012)
SPIT39	h^+ *leu1-32 ura4-DS/E ade6-210 otr1R::*$ura4^+$ *swi6Δ::kanMX6*	Iida (2006)
SPM3233	h^+ *leu1-32 ura4-DS/E ade6-210 otr1R::*$ura4^+$ *swi6::swi6**	This study
SPM3215	h^+ *leu1-32 ura4-DS/E ade6-210 otr1R::*$ura4^+$ *swi6::swi6Δ35-65*	This study
SPM3241	h^+ *leu1-32 ura4-DS/E ade6-210 otr1R::*$ura4^+$ *swi6::mini-swi6β*	This study
SPM3250	h^+ *leu1-32 ura4-DS/E ade6-216 otr1R::*$ura4^+$ *swi6::mini-swi6β cdc25-22*	This study
SPM3272	h^+ *leu1-32 ura4-DS/E ade6-210 otr1R::*$ura4^+$ *swi6::mini-swi6β-S12,13A*	This study
SPM3274	h^+ *leu1-32 ura4-DS/E ade6-210 otr1R::*$ura4^+$ *swi6::mini-swi6β-S12,13E*	This study
SPM4365	h^+ *leu1-32 ura4-DS/E ade6-210 otr1R::*$ura4^+$ *swi6::mini-swi6β-S12,13E cdc25-22*	This study
SPM3395	h^+ *leu1-32 ura4-DS/E ade6-210 otr1R::*$ura4^+$ *swi6::swi6Δ2-65*	This study
SPM3162	h^+ *leu1-32 ura4-DS/E ade6-210 otr1R::*$ura4^+$ *swi6::swi6Δ2-31*	This study
SPM3140	h^+ *leu1-32 ura4-DS/E ade6-210 otr1R::*$ura4^+$ *swi6::swi6-S12,13A*	This study
SPM3144	h^+ *leu1-32 ura4-DS/E ade6-210 otr1R::*$ura4^+$ *swi6::swi6-S12,13E*	This study
SPM4505	h^{90} *leu1-32 ura4-DS/E ade6-216 swi6::EGFP-*$swi6^+$	This study
SPM4511	h^{90} *leu1-32 ura4-DS/E ade6-216 swi6::EGFP-mini-swi6β*	This study
SPM4524	h^{90} *leu1-32 ura4-DS/E ade6-216 swi6::EGFP-mini-swi6β clr4Δ::hphMX4*	This study
AH1026	h^- *leu1-32 ura4-D18 ade6-216 swi6::EGFP-*$swi6^+$ *hht1::hht1-mCherry-kanMX6 cdc25-22*	This study
AH1024	h^- *leu1-32 ura4-D18 ade6-216 swi6::EGFP-swi6-S12,13A hht1::hht1-mCherry-kanMX6 cdc25-22*	This study
AH1025	h^- *leu1-32 ura4-D18 ade6-216 swi6::EGFP-swi6-S12,13E hht1::hht1-mCherry-kanMX6 cdc25-22*	This study
SPM4080	h^+ *leu1-32 ade6-210*	This study
SPM4100	h^+ *leu1-32 ade6-210 swi6Δ::hphMX4*	This study
SPM4081	h^+ *leu1-32 ade6-210 swi6::swi6-S12,13A*	This study
SPM4089	h^+ *leu1-32 ade6-210 swi6::swi6-S12,13A*	This study
SPM4067	h^+ *leu1-32 ade6-216 ark1-T8-GFP<<kanMX6*	This study
SPM4102	h^+ *leu1-32 ade6-216 ark1-T8-GFP<<kanMX6 swi6Δ::kanMX6*	This study
SPM4069	h^+ *leu1-32 ade6-216 ark1-T8-GFP<<kanMX6 swi6::swi6-S12,13A*	This study
SPM4083	h^+ *leu1-32 ade6-216 ark1-T8-GFP<<kanMX6 swi6::swi6-S12,13E*	This study
SPYB232	h^+ *leu1-32 ura4-DS/E ade6-216 otr1R::*$ura4^+$	Ishida (2012)
SPM4071	h^+ *leu1-32 ura4-DS/E ade6-216 otr1R::*$ura4^+$ *swi6::swi6-S12,13A*	This study
SPM4085	h^+ *leu1-32 ura4-DS/E ade6-216 otr1R::*$ura4^+$ *swi6::swi6-S12,13E*	This study
SPM4072	h^+ *leu1-32 ura4-DS/E ade6-216 otr1R::*$ura4^+$ *bir1-T1<<kanMX6*	This study
SPM4104	h^+ *leu1-32 ura4-DS/E ade6-210 otr1R::*$ura4^+$ *bir1-T1<<kanMX6 swi6Δ::kanMX6*	This study
SPM4073	h^+ *leu1-32 ura4-DS/E ade6-216 otr1R::*$ura4^+$ *bir1-T1<<kanMX6 swi6::swi6-S12,13A*	This study
SPM4087	h^+ *leu1-32 ura4-DS/E ade6-210 otr1R::*$ura4^+$ *bir1-T1<<kanMX6 swi6::swi6-S12,13E*	This study
SPM1468	h^- *leu1-32 ura4-DS/E ade6-210 otr1R::*$ura4^+$	This study
SPM4098	h^- *leu1-32 ura4-DS/E ade6-210 otr1R::*$ura4^+$ *swi6Δ::hphMX4*	This study
SPM4074	h^- *leu1-32 ura4-DS/E ade6-210 otr1R::*$ura4^+$ *swi6::swi6-S12,13A*	This study
SPM4075	h^- *leu1-32 ura4-DS/E ade6-210 otr1R::*$ura4^+$ *pic1-T296<<hphMX4*	This study
PR62	h^- *leu1-32 pic1-T296<<hphMX4 swi6Δ::*$ura4^+$	Kawashima (2007)
SPM4077	h^- *leu1-32 ura4-DS/E ade6-210 otr1R::*$ura4^+$ *pic1-T296<<hphMX4 swi6::swi6-S12,13A*	This study
SPM4092	h^- *leu1-32 ura4-DS/E ade6-210 otr1R::*$ura4^+$ *pic1-T296<<hphMX4 swi6::swi6-S12,13E*	This study

Supplementary Table S2. List of primers used in this study

Name	Sequence	Experiment
Bam-Nde-Swi6_Fw	5'-AGG ATC CCA TAT GAA GAA AGG AGG TGT TCG ATC T-3'	Cloning
Sal-Swi6_Rv	5'-TGT CGA CTT ATT CAT TTT CAC GGA ACG TTA-3'	Cloning
Ark1_Fw	5'-GGA TCC ATG TCA GAT TCA AAG TTG GCA GA-3'	Cloning
Ark1_Rv	5'-GAA TTC GGT TAG GAA GAT TCA GAA CTT TTG-3'	Cloning
Swi6_N_Rv	5'-GAA TTC TTA TTC TTT GGC ATT TTC TTT CAA-3'	Cloning
Swi6_CD_Fw	5'-ACA TAT GGA TGA ATA TGT TGT AGA AAA G-3'	Cloning
Swi6_CD_Rv	5'-GGA ATT CAT CCA TGT TCA TTC CAA T-3'	Cloning
Swi6_Hinge_Fw	5'-CAT ATG GAA CAT GGA GGA AGA CCA GAA CC-3'	Cloning
Swi6_Hinge_Rv	5'-GAA TTC TTA TTG TTT AAC CGT CAG CTC TCT-3'	Cloning
Swi6_CSD_Fw	5'-GGA TCC ACG GTT AAA CAA GTA GAA AAC TA -3'	Cloning
Swi6_S8A	5'-GGA GGT GTT CGA GCT TAT CGG CGC TCC TC-3'	Mutagenesis
Swi6_S12A	5'-ATC TTA TCG GCG CGC CTC TAC TTC AAA ACG-3'	Mutagenesis
Swi6_S13A	5'-TTA TCG GCG CTC CGC TAC TTC AAA ACG ATC-3'	Mutagenesis
Swi6_S12 13A	5'-TCT TAT CGG CGC GCC GCT ACT TCA AAA CGA -3'	Mutagenesis
Swi6_S12 13E	5'-TCT TAT CGG CGC GAA GAG ACT TCA AAA CGA TCA G-3'	Mutagenesis
Swi_S18A	5'-CTA CTT CAA AAC GAG CAG TTA TTG ACG ACG-3'	Mutagenesis
Swi6_S142A	5'-GAA GAC CAG AAC CTG CTA AAA GAA AGA GGA-3'	Mutagenesis
Swi6_T147A	5'-CTA AAA GAA AGA GGG CTG CAA GAC CTA AA-3'	Mutagenesis
Swi6_S159A	5'-GAA GCA AAG GAG CCT GCA CCA AAG TCT CGT-3'	Mutagenesis
Swi6_S162A	5'-AGC CTT CAC CAA AGG CTC GTA AAA CTG ATG-3'	Mutagenesis
Swi6_T165A	5'-AAA GTC TCG TAA AGC TGA TGA AGA TAA ACA-3'	Mutagenesis
Swi6_S224A	5'-CCT TCA CAA AAA GAA GCG AAT GAA AGT GAG-3'	Mutagenesis
Swi6_S246A	5'-CGA AAA AGA AGC CTG CTC CGG AAG TTC CCA-3'	Mutagenesis
Swi6_ΔN_Fw	5'-ACA AAG GAG GCT ATT GCT TCT CAT A-3'	Mutagenesis
Swi6_ΔN_Rv	5'-CAT TTT TCA CTT GTC TTA ATA TGA A-3'	Mutagenesis
Swi6_Δ2-17_Fw	5'-AGG ATC CCA TAT GTC AGT TAT TGA CGA CGA TTC GGA-3'	Mutagenesis
Swi6_Δ2-31_Fw	5'-ACA TAT GAT GAC AAA GGA GGC TAT TGC T-3'	Mutagenesis
Swi6_Δ2-65_Rv	5'-ACA TAT GAA TGC CAA AGA AGA AGA AGG AGG A-3'	Mutagenesis
HP1β_Hinge_Fw	5'-GTC GCG AAA AAC AGC ACA TGA GAC AGA TAA-3'	Domain swapping
HP1β_Hinge_Rv	5'-CGT TTA AAC CCT CGT GGC TTT TCT GAC TCT-3'	Domain swapping
ura4-RT-Fw1	5'-GGC CTC AAA GAA GTT GGT TTA CC-3'	RT-qPCR
ura4-RT-Rv1	5'-GAA GAC ATT TCA GCC AAA AGC A-3'	RT-qPCR
Cen_dg_Fw	5'-CTG CGG TTC ACC CTT AAC AT-3'	ChIP-qPCR
Cen_dg_Rv	5'-CAA CTG CGG ATG GAA AAA GT-3'	ChIP-qPCR
act1_RT_Fw	5'-CGT GCC CCT GAA GCT CTT T-3'	ChIP-qPCR
act1_RT_Rv	5'-CTC ATG AAT ACC GGC GTT TTC-3'	ChIP-qPCR
